# Chirality-Dependent Supramolecular Biomaterials Remodeling of Scar Microenvironment via Integrin-Mediated Regulation for Hypertrophic Scars Therapy

**DOI:** 10.1007/s40820-026-02180-1

**Published:** 2026-04-24

**Authors:** Xueqian Wang, Chengyao Han, Hongrui Shan, Jinjin Li, Beibei Wu, Yixin Zhang, Ke Li, Chuanliang Feng

**Affiliations:** 1 State Key Lab of Metal Matrix Composites, Shanghai Key Laboratory for Molecular Engineering of Chiral Drugs, School of Materials Science and Engineering, Shanghai Jiao Tong University, Shanghai, 200240 People’s Republic of China; 2 Department of Plastic and Reconstructive Surgery, Shanghai Ninth People’s Hospital, Shanghai Jiao Tong University School of Medicine, Shanghai, People’s Republic of China; 3 Research Center of Precision Sensing and Control, Institute of Automation, Chinese Academy of Sciences, Beijing, 100190 People’s Republic of China; 4 National Key Laboratory of Advanced Micro and Nano Manufacture Technology, Shanghai Jiao Tong University, Shanghai, 200240 People’s Republic of China

**Keywords:** Chirality, Biomaterial, Hypertrophic scars, Integrin, Fibroblasts

## Abstract

Hypertrophic scars, characterized by excessive fibroblast activation, present significant clinical challenges. Current treatments (e.g., laser, surgery, steroids) face limitations: Surgery is costly and associated with high recurrence rates, while pharmacological interventions often induce pain and exhibit low bioavailability or efficacy. To address this, we engineered a novel chiral supramolecular biomaterial derived from L-/D-phenylalanine and D-phenylalanine (L/DP) with well-defined nanostructure and optical activity. L/DP achieved biomimetic integration and stereoselective regulating of integrin β1 (ITGβ1) in scar tissue. In vitro, LP suppressed fibroblast proliferation by downregulating ITGβ1 (72%), inhibiting FAK/PI3K/AKT signaling and TGF-β1. In vivo (rabbit ear HS model), LP reduced scar thickness (54%), collagen deposition (39%), and α-SMA expression (45%), outperforming conventional drugs by 23%. This chirality-directed strategy provides a drug-free, painless, and highly effective HS therapy via integrin-mediated remodeling of the scar microenvironment and holds substantial clinical promise.

**Supplementary Information:**

The online version contains supplementary material available at 10.1007/s40820-026-02180-1.

## Highlights

- A chiral supramolecular biomaterial (L-/D-phenylalanine and D-phenylalanine, L/DP) is rationally designed with defined chiral nanostructure and optical activity for hypertrophic scars (HS) therapy.
- The LP biomaterial significantly inhibited excessive fibroblast proliferation by downregulating ITGβ1 by 72%, suppressing downstream FAK/PI3K/AKT signaling and TGF-β1 activation.
- In rabbit HS models, LP outperforms drug and DP, reducing scar thickness by 54%, collagen deposition by 39%, and α-SMA expression by 45%, offering an effective, painless, drug-free HS therapy strategy.

**Supplementary Information:**

The online version contains supplementary material available at 10.1007/s40820-026-02180-1.

## Graphical abstract

**Figure:**
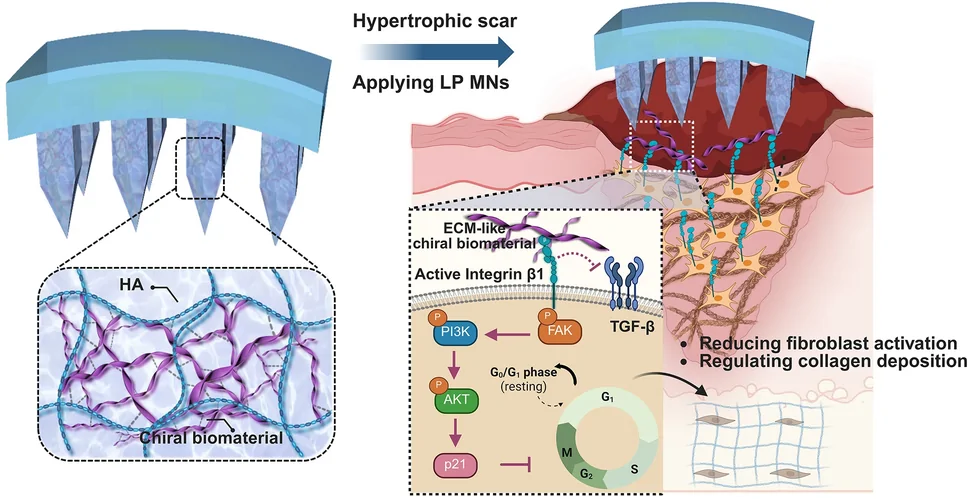

## Introduction

Hypertrophic scars (HS), a common consequence of aberrant skin wound healing, are characterized by excessive fibroblast proliferation, disorganized collagen deposition, and persistent inflammation [1–3]. Unlike physiological scars, HS exhibit sustained proliferative capacity without spontaneous regression, resulting in functional impairment (e.g., joint contracture), aesthetic disfigurement, and intractable pruritus, which severely compromise patients’ physical and psychological well-being. Epidemiological studies indicate that HS develop in 60%–80% of burn patients and 30%–50% of postsurgical cases, with global annual healthcare costs exceeding US$12 billion and quality-of-life reductions of up to 40%, underscoring the urgent need for more effective therapies [4–6].

Current interventions (e.g., laser therapy, surgery, steroid injections, and pharmacological agents) have critical limitations [7]. Corticosteroids, the first-line pharmacological option, have limited bioavailability (< 15%) in dense scar tissue because extracellular matrix (ECM) barriers impede drug diffusion and are associated with adverse events (e.g., skin atrophy, hypopigmentation) and poor patient compliance [8, 9]. Laser therapy, relying on thermal ablation, requires 5–8 sessions, fails in deep/mature scars, and carries long-term pigmentation risks [10]. Surgical resection, although immediately effective in reducing scar volume, frequently triggers aberrant wound healing, with recurrence rates of up to 70% within 12 months and a consequent need for repeated procedures [11–13]. Other pharmacological approaches (e.g., anti-TGF-β agents) face additional challenges, including poor penetration into scar tissue, off-target toxicity, and limited durability of response due to redundant signaling networks [14–16]. Critically, these treatments primarily rely on non-specific anti-inflammatory effects or mechanical disruption and therefore lack targeted regulation of the fibroblast-driven profibrotic microenvironment, highlighting the need for innovative therapeutic strategies [17, 18].

To address this therapeutic gap, it is essential to regulate key regulators of fibroblast activation and the profibrotic microenvironment [19–21]. Among these, the mechanosensitive integrin β1 (ITGβ1) has emerged as a master regulator orchestrating fibrotic signaling [22–25]. However, conventional integrin inhibitors (e.g., RGD peptides) are limited by poor spatiotemporal control and insufficient ECM specificity [26–30]. Advances in biomaterials engineering provide new therapeutic opportunities. Biomaterials designed to recapitulate essential features of native tissues can interface with cells in a context-specific manner to modulate pathological signaling [31–34]. For example, gelatin-based cryogels with interconnected porous nanostructures regulate cell behavior via bioactive motifs [35–37], whereas hyaluronic acid derivatives functionalized with hydrophobic chains or catechol groups target cell membrane receptors through synergistic physicochemical interactions [38]. Such biomaterials enhance specificity and durability by integrating topographical cues with biochemical targeting, and their ECM-mimetic architectures can guide ordered tissue regeneration, thereby mitigating the disorganized collagen deposition characteristic of HS.

**Fig. 1:**
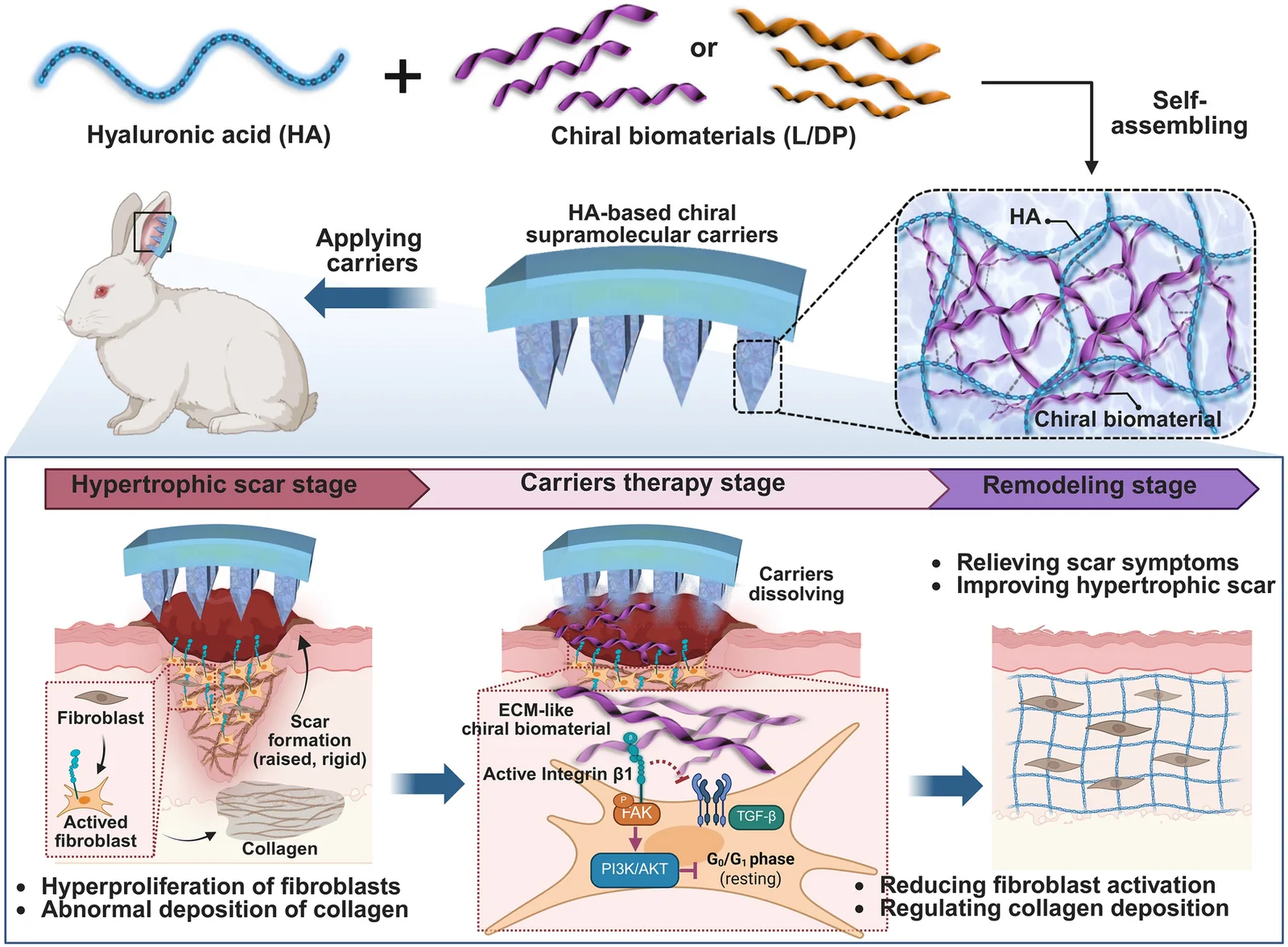
Schematic of hypertrophic scars treatment by chiral supramolecular biomaterial via regulating integrin β1 and integrin-mediated pathways. Schematic illustration of carriers (MNs) consisting of HA and chiral supramolecular biomaterial, scar formation, and regulating hypertrophic scars mechanisms. HA, hyaluronic acid

Herein, we rationally designed a chiral supramolecular biomaterial (L/DP) with well-defined chiral nanostructures and optical activity, enabling stereoselective interaction with integrin β1 (ITGβ1) within scar tissue (Fig. 1). To validate its therapeutic potential, we systematically conducted both in vitro and in vivo evaluations. In vitro, L/DP inhibited fibroblast hyperactivity by downregulating ITGβ1 expression by 72% and suppressing FAK/PI3K/AKT signaling and TGF-β activation. To enhance tissue penetration, L/DP was further incorporated into a hyaluronic acid-based carrier for localized, noninvasive delivery. In vivo, LP reduced scar thickness (54%), collagen deposition (39%), and α-SMA expression (45%), outperforming conventional therapies by 23%. This work introduces a therapeutic biomaterial that combines drug-free intervention, integrin regulation, and carrier-enabled noninvasive delivery, collectively achieving superior efficacy to conventional HS therapies.

## Experimental Section

### Materials

All the materials used in this study can be found in Supplementary Information.

### Synthesis and Mechanism of L/DP Biomaterial

The detailed synthetic methods for L/DP are described in Supplementary Information. Incorporating phenylalanine into chiral supramolecular biomaterials enhances biosafety, leveraging its inherent chirality (with L- and D-configurations) to impart chiral characteristics to the material. Formation of such chiral supramolecular biomaterials relies on a delicate balance between dissolution and aggregation in aqueous media, where non-covalent interactions act as key assembly drivers. Within the designed chiral biomaterial, the benzene ring of phenylalanine mediates critical hydrophobic interactions for self-assembly. Meanwhile, the amide group—formed via condensation of phenylalanine's amino group with 1,4-benzenedicarbonyl chloride—enables hydrogen bonding during assembly. Introduction of ethylene glycol chains at the C-terminus further improves hydrophilicity, resulting in a C2-symmetric architecture. In this structure, the central hydrophobic benzene ring promotes aqueous aggregation, while flanking amide groups strengthen assembly via hydrogen bonding. Peripheral phenylalanine benzene rings provide additional hydrophobic stabilization to the supramolecular framework. The outermost hydrophilic ethylene glycol chains remain solvated freely in water, effectively inhibiting over-aggregation and precipitation caused by excessive hydrophobicity in intermediate assemblies.

### Preparation of L/DP Biomaterial

L/DP (0.5 mg mL^−1^, abbreviated as 0.5% L/DP) was firstly added into deionized water and uniformly dispersed by ultrasound. Then, the solution was heated to 90 °C and maintained for 10 min under continuous stirring until a transparent solution was then cooled to room temperature (25 °C) to obtain L/DP. Similarly, 1% L/DP (abbreviated as 1% L/DP), 2% L/DP (abbreviated as 2% L/DP), and 3% L/DP (abbreviated as 3% L/DP) were prepared by changing the concentration of L/DP.

### Characterization of LP and DP Biomaterials

The synthesis of LP and DP molecules was verified by 1H-NMR and mass spectrometry. The surface morphology LP and DP biomaterials was investigated by a FM-SEM (field emission scanning electron microscope, FEI Quanta 250). All samples were diluted by DI water and dripped onto silicon wafers before testing. After drying, the surface of samples was sputter coated with Au plating and examined on FM-SEM. Circular dichroism (CD) spectroscopy was utilized to analyze the optical property of LP and DP biomaterials. Briefly, the test was performed using the CD spectrometer (JASCO J-1500, Japan) in the 190–500 nm range using a quartz cuvette with 0.2 mm optical diameter at N_2_ (nitrogen) atmosphere.

### Cell Cultures

Primary hypertrophic scar fibroblasts (HSFs) were isolated from human pathological hypertrophic scar tissues, provided by the Department of Plastic and Reconstructive Surgery in the Shanghai Ninth People’s Hospital, Shanghai, China. In brief, the excised skin tissue was cleaned with sterile phosphate-buffered saline (PBS) and subcutaneous attached fat was removed. After that, the scar tissue was cut into small pieces and incubated continuously with Type I collagenase (0.2% w/v) and pancreatin (0.25%) at 37 °C for 20 min. Cells were liberated from enzyme-digested tissue and cultured in high-glucose Dulbecco’s modified Eagle’s medium (DMEM; Gibco). The isolated cells were characterized by immunofluorescence staining, showing positive expression for α-SMA. All cells were incubated in an incubator (37 °C, 5% CO_2_). All experiments were conducted using HSFs from the 3rd to 5th passage, and cell purity was confirmed to be > 90% prior to use.

### Study of Cell Viability, Proliferation, and Migration In Vitro

At the beginning, all the cell plates were uniformly coated with different concentrations of L/DP hydrogels. To assess the cytotoxicity of chiral hydrogels to HSFs, a live/dead assay was initially performed. The HSFs were seeded on 24-well plates (1 × 10^5^ cells per well) and cultured for 48 h. Afterward, the cells were stained using calcein-AM (2 μM) and PI (4 μM) for 20–40 min. The fluorescence images of live/dead staining were visualized by a fluorescence microscope (Olympus IX73). The HSFs were seeded into 96-well plates (1 × 10^4^ cells per well) and incubated for various times (24, 48, and 72 h). Subsequently, Cell Counting Kit 8 assay (CCK-8, Dojindo) was performed at indicated times according to the standard protocol and the optical density (OD) value at 450 nm was measured on a microplate reader (TECAN Infinite 200 PRO). The EdU kit (Kaiji Biotech.) was also performed to evaluate the proliferation rate of the HSFs following the instruction manuals. Briefly, the HSFs were incubated in 96-well plates (5 × 10^3^ cells per well) for 24 h. Afterward, cells were labeled with EdU (10 mM) in prewarmed growth medium and incubated for 2 h. The cells were then fixed in 4% paraformaldehyde (PFA) for 30 min, followed by incubation with Apollo Mixture and stained with DAPI. The cells were observed using a fluorescence microscope (Olympus IX73) and the EdU-positive cells were counted by Image J V. 1.52 (NIH, USA). The scratch assay was performed on the HSFs to assess cell migration. The HSFs were first seeded at a density of 1 × 10^6^ cells per well in 6-well plates. When cells formed a monolayer, 200-μL pipette tips were used to scratch five parallel lines in each well and gently washed with PBS to remove the accumulated cell debris. At desired time points (0, 12, and 24 h), cells were photographed with an Olympus microscope (DP73, JPN). The areas of scratch were analyzed by Image J V. 1.52 (NIH, USA) and calculated using Eq. (S1).

### Evaluation of Cellular Morphology

For observation of cellular morphology, the HSFs were firstly seeded onto 12-well plates coated with hydrogels at a density of 1 × 10^5^ cells/well for 48 h. Following that, cells were visualized using a fluorescence microscope (Olympus IX73). And for further analysis of the change of cell morphology, the cells were fixed with 4% paraformaldehyde (PFA) for 20 min and stained with Phalloidin-iFluor 488 reagent according to the standard instruction. The images of cell were photographed by a fluorescence microscope (Olympus IX73).

### RNA Sequencing

The HSFs were seeded onto 6-well plates coated with hydrogels (1 × 10^6^ cells/well) and cultivated for 48 h in vitro. Subsequently, the RNA mini kit (Qiagen, Germany) was utilized to extract the total RNA from HSFs following various treatments (*n* = 3 independent biological replicates per group). The total amount of RNA was precisely quantified with Qubit (Thermo, Waltham, MA, USA), and the RNA integrity was examined by agarose gel electrophoresis. All samples were ensured to have an RNA integrity number (RIN) ≥ 8.0 and a 28*S*/18*S* RNA ratio ≥ 1.5. After constructing strand-specific libraries with the TruSeq RNA sample preparation kit (Illumina, San Diego, CA, USA), sequencing was performed on the Illumina Novaseq 6000 instrument by the commercial service of Genergy Biotechnology Co. Ltd. (Shanghai, China) and the quality of the raw sequencing data was evaluated by FastQC software. Gene expression was measured utilizing the fragments per kilobase of exon model per million mapping reads (FPKM) approach. The DESeq algorithm was adopted to calculate the differentially expressed genes with fold changes (FC) > 2 and *p* < 0.05. The enrich *R* package was applied to perform GO and KEGG pathway analyses (Kyoto Encyclopedia of Genes and Genomes, http://www.genome.ad.jp/kegg). GSEA was evaluated using the GSEA analysis tool (http://www.broadinstitute.org/gsea/index.jsp) and Hallmark dataset.

### Flow Cytometry for Cell Cycle and Cell Apoptosis Analysis

The HSFs were seeded onto 6-well plates coated with hydrogels at 1 × 10^6^ cells/well and growth in complete media for 24 h. For cell cycle analysis, the cells were digested with 0.25% pancreatin, collected by centrifugation, and fixed with cooled 70% ethyl alcohol. Then, cells were rinsed with PBS and incubated with propidium iodide/ribonuclease (PI/RNase) solution for 1 h in the dark. The proportion of cells in the cell cycle (*G*0, *G*1, *S*, and *G*2) was quantified by flow cytometry (CytoFLEX LX, Beckman Coulter). For cell apoptosis detection, the cells were stained with FITC–annexin V and propidium iodide (PI) according to the standard protocol and determined by flow cytometry (CytoFLEX LX, Beckman Coulter). All data were analyzed using FlowJo software (Treestar, USA).

### Western Blot Analysis

After treatment, total proteins were extracted from HSFs using the RIPA lysis buffer (Beyotime, China) and quantified using the BCA kit (Beyotime, China) according to the manufacturer’s instructions. Then, the protein samples were resolved through sodium dodecyl sulfate–polyacrylamide gel electrophoresis (SDS-PAGE) and transferred to poly(vinylidene fluoride) (PVDF) membranes (Millipore, USA). Following blocking with 5% non-fat dried milk for 2 h, the membranes were incubated with particular primary antibodies including PCNA (1:1000), cyclin D1(1:10000), p21(1:1000), ITGβ1(1:1000), Vinculin(1:5000), FAK(1:1000), p-FAK(1:1000), AKT(1:5000), p-AKT(1:1000), PI3K(1:1000), p-PI3K(1:1000), β-actin(1:50000), and GAPDH(1:5000) overnight at 4 °C. Subsequently, these PVDF membranes were incubated with corresponding secondary antibodies (1:5000) for 1 h at room temperature. The membranes were rinsed again with Tris-buffered saline with Tween-20 (TBST) and were visualized using a gel imaging system (Fusion FX7, France). The categories of all reagent used in this study are shown in Table S2.

### Immunofluorescence Staining

The HSFs were fixed with 4% paraformaldehyde (PFA) for 30 min and permeabilized with 0.1% Triton X-100 in PBS for another 20 min at room temperature. After being blocked with 5% bovine serum albumin (BSA) for 30 min, the cells were incubated with indicated primary antibody at 4 °C overnight. The following day, the cells were recognized with fluorescent-labeled secondary antibodies for 1 h and cell nuclei were labeled with DAPI. The images were captured with a laser scanning confocal microscope (Leica SP8 STED, German).

### Classic Molecular Dynamic Simulation

Materials Studio was used to build models of LP and DP molecules. The density functional theory (DFT) calculations were performed using the Gaussian 16 software. The B3LYP functional was adopted for all calculations in combination with the D3BJ dispersion correction. For geometry optimization, def2tzvp basis set was used for all atmos. Then, Multiwfn was used to fit the restrained electrostatic potential (RESP) charge. Other molecules bond and nonbond parameters were obtained via AuToFF software. The atomic structures of ITGβ1 protein (RCSB ID: 8RPQ) were sourced from the Protein Data Bank (8RPQ). All-atom molecular dynamic (MD) simulations were conducted using the GROMACS software package, version 2021.5. The Amber03 force field was employed to describe the molecule. SPCE force field was used for water molecules. Firstly, we arranged 91 LP molecules and 91 DP molecules in an orderly manner and placed them at the bottom of a simulation box with dimensions of 9 nm × 9 nm × 10 nm. Then, ITGβ1 were placed within a range of 1 nm above them. Subsequently, the entire simulation box was filled with water molecules, and the system’s charge was neutralized using Cl- ions to construct the simulation system. Energy minimization was initially carried out for systems, employing the steepest descent method to address initial contact issues. Followed by a short simulation of 1 ns using the NPT system to make the box fully compressed. Subsequently, a 100-ns simulation was then performed under the NPT ensemble to relax the structure and achieve system equilibrium. During the entire simulation process, the base layer composed of LP and DP was subjected to positional constraints. The pressure was maintained at *P* = 1.0 bar using a Parrinello–Rahman barostat, and the temperature was controlled at 300 K using a velocity-rescale thermostat with a coupling constant of *τ* = 0.1 ps. Nonbonded interactions were computed with a cutoff of 1.2 nm, and long-range electrostatic interactions were computed using the particle-mesh Ewald summation method. All hydrogen bonds were constrained using the LINCS algorithm. Simulations were performed with a time step of 2 fs, and the neighbor list was updated every 10 steps. Periodic boundary conditions were applied in all three directions. PyMOL-3.0.3 (http://www.pymol.org/pymol) was used for visualization.

### Fabrication of HA-Based Chiral Supramolecular Carriers

The HA-based chiral supramolecular carriers (L/D-HA MNs) were fabricated using a polydimethylsiloxane (PDMS) micromold with the base diameter and height of each needle cavity being 320 and 1000 μm, respectively. These needle cavities were arranged in a 15 × 15 array with a tip-to-tip distance of 800 μm. To fabricate the L/D-HA MNs, 200 μL well-mixed pregel solutions of L/DP (3 mg mL^−1^) and oligo-HA (20 wt%) were first deposited on the PDMS micromold. Then, the micromold was vacuumed with negative pressure to remove air bubbles and centrifugated at 4000 rpm for 6 min to force the mixture into the needle voids. This process will be repeated several times to ensure that the needle tip is fully filled with pregel solutions. Subsequently, the micromold containing the mixture was kept in a desiccator at room temperature overnight. After complete desiccation, the L/D-HA MNs patch was carefully detached from the PDMS micromold. For the fabrication of blank HA MNs, no L/DP was added into the oligo-HA solution. A silicon sheet with a thickness of 0.3 mm was attached to the back of the MNs using waterproof epoxy resin (KE-45-W, Shin-Etsu Chemical Co. Ltd.).

### Characterization of L/D-HA MNs

The morphology of L/D-HA MNs was investigated by field emission scanning electron microscope (FM-SEM, FEI Quanta 250), stereoscopic microscope (M125, Leica), and fluorescence microscope (Olympus IX73), respectively. To further visualize the L/D-HA MNs, Rhodamine dye was added when the microneedles were prepared and observed under fluorescence microscope (Olympus IX73).

### Mechanical Strength Test

The mechanical properties of the L/D-HA MNs and HA MNs were tested by a universal testing machine (Zwick/Roell Z020). The sample was fixed onto an aluminum plate with microneedle tips facing upward. A vertically directional force was applied to sample by a flat-headed stainless steel cylindrical probe at a constant displacement speed of 0.01 mm s^−1^. The data were measured until a predetermined displacement of 600 μm was attained. Then, the displacement distance was plotted against the compression force per needle and the displacement curve was recorded along with the load.

### Skin Insertion Ability of L/D-HA MNs In Vitro

To assess the insertion ability and insertion depth of L/D-HA MNs in vitro, L/D-HA MNs was loaded with Rhodamine dye and applied to the isolated rat tail skin for 1 min. After the MNs was removed, the micro-sized holes on skin were examined and rebuilt three-dimensional images by a laser scanning confocal microscope (Leica SP8 STED, German).

### Skin irritation Test of L/D-HA MNs in Rabbit Ear Skin

According to the existing experimental evaluation methods of skin irritation, the skin irritation response scores of each animal were as follows: no erythema (0 points), barely erythema (1 point), moderate erythema (2 points), severe erythema (3 points), purplish red spots with eschar formation (4 points), no edema (0 points), barely visible edema (1 points), visible edge of edema higher than the surrounding skin (2 points), skin bulge 1 cm clear outline (3 points), and edema bulge more than 1 cm and expanded scope (4 points). Stimulus response score was performed to calculate the average score. A multi-dose rabbit ear skin stimulation experiment was performed; that is, 12 rabbits were randomly divided into four groups and given HA MNs, L-HA MNs and D-HA MNs at the same location in ear skin as the experiment once a day, and the skin stimulation response is observed 1, 24, and 72 h after the removal of the MNs. Erythema formation integral = total erythema integrals/umber of animals; edema formation integral = total edema integral/number of animals.

### Animals and Human Tissues

New Zealand white rabbits (male, 1–3 kg) were obtained purchased from Shanghai Jiagan Biotechnology Co. Ltd (Shanghai, China). All animal tests were performed following protocols approved by the Ethical Committee for Animal Experiments of Shanghai Jiao Tong University (China, A2022048). All the human skin tissues were originated and approved by the Ethical Committee for Clinical Experiments of Shanghai Ninth People’s Hospital (China, SH9H-2023-TK285-1).

### Establishment of Rabbit Ear Hypertrophic Scar Model

To establish rabbit ear hypertrophic scar model, New Zealand white rabbits were initially anesthetized with a marginal ear vein injection of pentobarbital sodium (30 mg kg^−1^) and a supplemental dose of pentobarbital sodium was administered as necessary to maintain surgical anesthesia. After disinfection, four full-thickness excisional circular wounds (10 mm in diameter) were made on the ventral side of the rabbit ear and each wound was spaced more than 10 mm apart. Then, the majority of the perichondrium were removed by scalpel and the ear cartilage was retained. Thirty days after surgery, the wound completely healed and hypertrophic scars were formed accompanied with tissue eminence.

### Evaluation of L/D-HA MNs on HS In Vivo

The therapeutic efficacy of various HA-based chiral supramolecular carriers was evaluated in the rabbit ear hypertrophic scar model. The positive control group received pirfenidone (PFD) solution by HA MNs injection; the normal group was administered with PBS. For the carrier group, L-HA MNs and D-HA MNs were inserted into the HS tissues. The treated skin area was the same in each group, and administration was maintained for 30 days. Morphological changes of the treated skin area were recorded by photographing.

### Histological and Immumohistochemical Analysis

For histological assays, scar tissues from rabbit ear were collected at day 30. To make histological sections, the samples were firstly fixed in PFA (4%) and then decalcified in EDTA (10%, ethylenediaminetetraacetic acid). Further, the sections were visualized by hematoxylin and eosin (*H*&*E*) for pathological evaluation, Masson staining for collagen deposition analysis, and Sirius red staining for collagen fibrosis study. Based on H&E images, the scar elevation index (SEI) was quantified by Image J V. 1.52 and was calculated by Eq. (S2). For immumohistochemical staining, the sections were stained with antibodies to p-FAK, MMP-3, Vinculin, and α-SMA, respectively, according to the standard procedure (Thermo, USA). The stained slides were photographed with a fluorescence microscope (80i, Nikon).

### Quantitative Real-Time PCR Measurement

Scar tissues were harvested from rabbit ears on day 30. The total RNA was extracted from tissues using Trizol reagent (Solarbio, China) following the standard instructions. Next, the total extracted RNA was converted to cDNA (complementary DNA) by using a cDNA synthesis kit (TransScript®, TransGen Biotech, China). Finally, the quantitative PCR evaluation was performed by QuantStudio 1 Real-Time PCR system (ABI, Thermo Fisher, USA). Primer sequence of beta-actin (β-actin, housekeeping gene), glycogen synthase kinase-3 beta (GSK-3β), transforming growth factor-β (TGF-β), phospho-Smad3 (Thr179, p-Smad3), phospho-Smad2 (Ser250, p-Smad2), and beta-catenin (β-catenin) are listed in Table S3. The relative gene expression was calculated using the − 2^ΔΔCt^ method.

### Western Blotting Assay

Scar tissues at day 30 were collected and were gently washed three times with cold TBS to remove blood. Then, the samples were cut into small pieces and processed at 4 °C in a homogenizer using the RIPA lysate buffer. The supernatant was recovered after centrifugation at 12,000× g for 10 min, and the total protein concentration was quantified using the BCA assay kit. Equal amounts of extracted proteins samples were resolved by using gel electrophoresis and then transferred onto polyvinylidene difluoride (PVDF) membranes. After that, the membrane was incubated overnight with a specific primary antibody at 4 °C and an appropriate secondary antibody for 60 min at room temperature (RT). The membrane was rinsed three times with TBST and imaged on a gel imaging system. β-actin was used as control. Full information on reagents used in this study is listed in the Supplementary Information.

### Statistical Analysis

All experimental data were presented as the mean values ± standard deviation (SD). Statistical analysis was used to compare the differences between groups with t test (2-tailed) using the Prism (version 6, GraphPad Software), where the statistical significance was assigned as *p < 0.05, **p < 0.01, ***p < 0.001, and ****p < 0.0001, and ns (not significant versus the indicated group), respectively. For experiments with a single independent variable, one-way ANOVA was used to determine significant differences between groups, where the statistical significance was defined as *p < 0.05, **p < 0.01, ***p < 0.001, and ****p < 0.0001, respectively.

## Results and Discussion

### Preparation and Characterization of L/DP and L/D-HA MNs

**Fig. 2:**
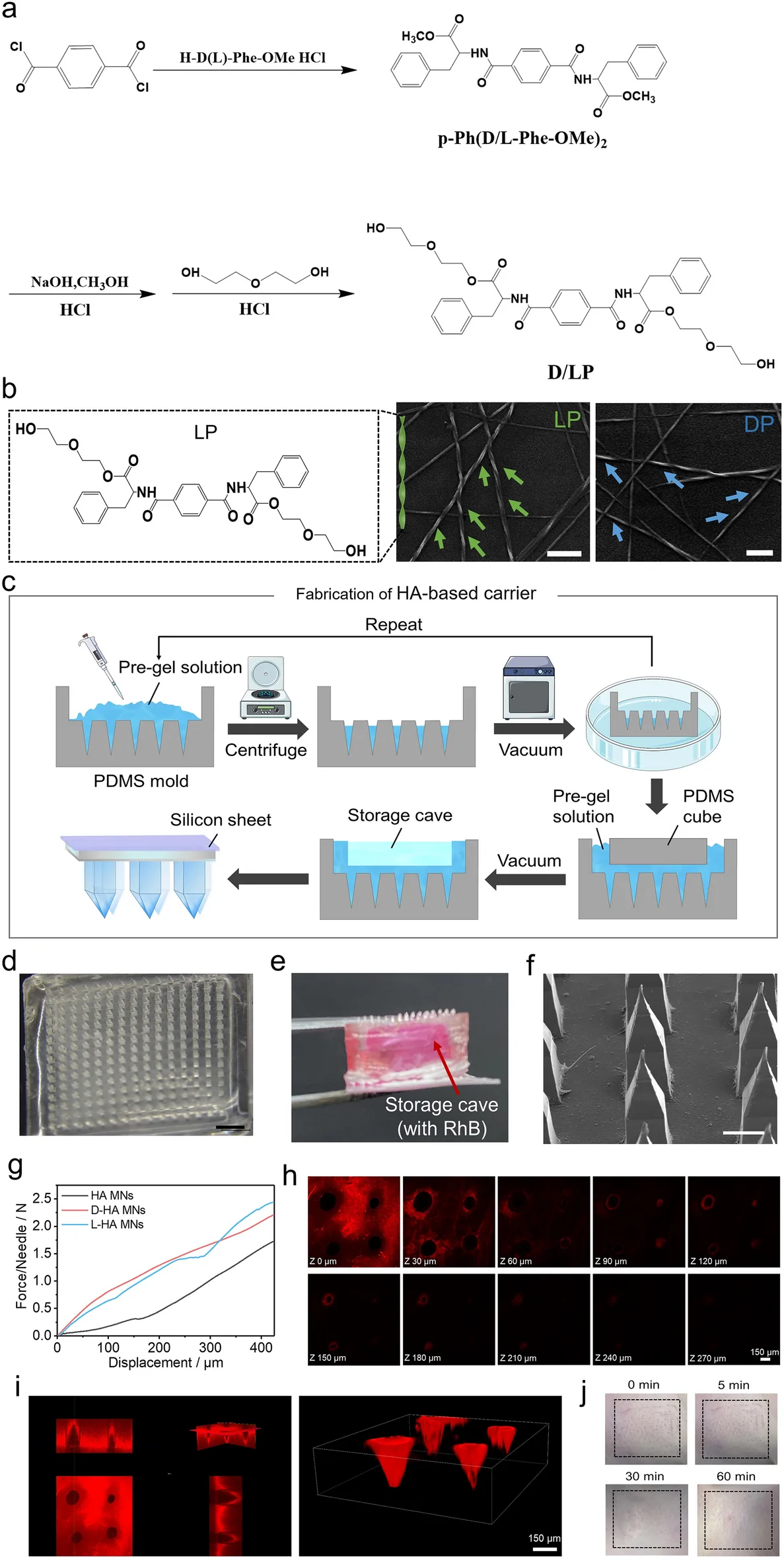
Preparation and characterizations of chiral supramolecular biomaterials and HA-based chiral supramolecular carriers. **a** Synthesis procedures of L/DP. **b** Molecular structure of LP and representative SEM images of LP and DP. Scale bar, 500 nm. **c** Schematic of the fabrication process of storage MNs. **d** Representative photographs of L-HA MNs. Scale bar, 1 mm. **e** Photograph of L-HA MNs loaded with Rhodamine B solution at the backing layer. **f** The SEM images of L-HA MNs. Scale bar, 500 μm. **g** Mechanical strength of HA MNs, L-HA MNs, and D-HA MNs. **h** Representative confocal images of Rhodamine B-labeled L-HA MNs from a bottom view. The intervals at *z*-direction were set as 30 μm. Scale bar, 150 μm. **i** Reconstruction images of L-HA MNs. Scale bar, 150 μm. **j** Representative photographs of L-HA MNs traces on living rabbit ear skin

Chiral molecules derived from *L*- and *D*-phenylalanine amphiphiles were synthesized by a conventional solution-phase method (Figs. 2a and S1) [39, 40]. The ^1^H-NMR spectroscopy and mass spectrometry confirmed the successful synthesis of the chiral molecules (Figs. S2–S5). In deionized water, these molecules self-assembled into supramolecular structures (abbreviated as LP and DP, respectively) via a heating–cooling process, with a critical assembly concentration of 0.5 mg mL^−1^ (Fig. S6). Scanning electron microscopy (SEM) revealed distinct chiral nanostructures: LP formed left-handed helical fibers, whereas DP assembled into right-handed nanofibers. Quantitative analysis showed negligible differences in their structural parameters: LP fibers exhibited a diameter of 150.09 ± 1.56 nm and a helical pitch of 470.14 ± 1.20 nm, versus 149.94 ± 1.69 nm and 465.05 ± 2.03 nm for DP (Figs. 2b, S7, and S8). Despite their opposite handedness, LP and DP therefore possessed highly similar helical parameters (Figs. 2b, S7, and S8). Circular dichroism (CD) spectroscopy further confirmed their chiral optical activity: LP displayed positive and negative Cotton effects at approximately 227 and 278 nm, respectively, whereas DP exhibited mirror-symmetric signals at approximately 217 and 275 nm (Fig. S9). Collectively, these data demonstrate that *L*/DP, with nanoscale diameters, well-defined helical morphologies, and pronounced chiral optical activity, closely recapitulate key structural features of the extracellular matrix (ECM). In particular, the helical pitch of these nanofibers (approximately 465–470 nm) mimics the hierarchical periodicity observed in certain ECM components; for example, collagen fibrils exhibit a characteristic banding pattern with a periodicity of ~ 67 nm, and higher-order helical arrangements in ECM fibers contribute to their mechanical resilience and biological functionality [41]. This structural similarity suggests that LP and DP nanofibers can provide a biomimetic microenvironment analogous to native ECM, which is critical for modulating cellular behavior.

To enhance delivery into stiff scar tissue, hyaluronic acid (HA)-based carriers (HA MNs) loaded with chiral biomaterials (abbreviated as L-HA MNs or D-HA MNs) were fabricated using a PDMS micromold (Fig. 2c) [42–47]. Stereomicroscopy and SEM revealed that the L-HA MNs consisted of a 15 × 15 array of needles (320 μm base diameter, 1000 μm height) with a rear storage cavity (capacity up to 1 mL) (Fig. 2d–f). Both HA MNs and D-HA MNs displayed identical morphological characteristics (Figs. S10 and S11). To further visualize the MNs, Rhodamine B (RhB) was added, and fluorescence micrographs confirmed successful MN fabrication, with each needle remaining intact (Fig. S12). For effective transdermal delivery, the carrier must possess sufficient mechanical strength to pierce the skin and reach deep lesion sites. Previous reports indicate that a fracture force of 0.1–0.2 N per needle is required to penetrate the stratum corneum [48]. Mechanical testing showed that L-HA MNs and D-HA MNs generated approximate forces of 2.5 and 2.2 N per needle, respectively, which is sufficient to penetrate scar tissue (Fig. 2g). Furthermore, in vitro penetration assays using RhB-labeled L-HA MNs demonstrated a penetration depth of ~ 270 μm in rat tail skin, and three-dimensional reconstruction confirmed that needle morphology remained intact after insertion (Fig. 2h, i). In vivo, the puncture marks gradually faded and were almost undetectable 60 min after application (Fig. 2j). No discernible irritation reactions (edema, erythema, etc.) were observed on the skin up to 72 h post-application, indicating minimal invasiveness and good local tolerability of the carrier (Fig. S13 and Table S1). Regarding the metabolic fate of the *D*-phenylalanine-based nanofibers, it is important to note that these structures are assembled via reversible non-covalent interactions. Unlike covalent polymers that require bond cleavage for degradation, supramolecular assemblies undergo disassembly when the local concentration drops below the critical aggregation concentration (CAC) due to diffusion and fluid exchange in the dermis. Although the *D*-amino acid components are resistant to proteolysis, the disassembled monomers are sufficiently small (< 1000 Da) to be cleared via the lymphatic and circulatory systems and excreted renally, thereby avoiding long-term accumulation or granuloma formation.

### L/DP Regulates ITGβ1 and ITGβ1-Mediated Signaling Pathways

**Fig. 3:**
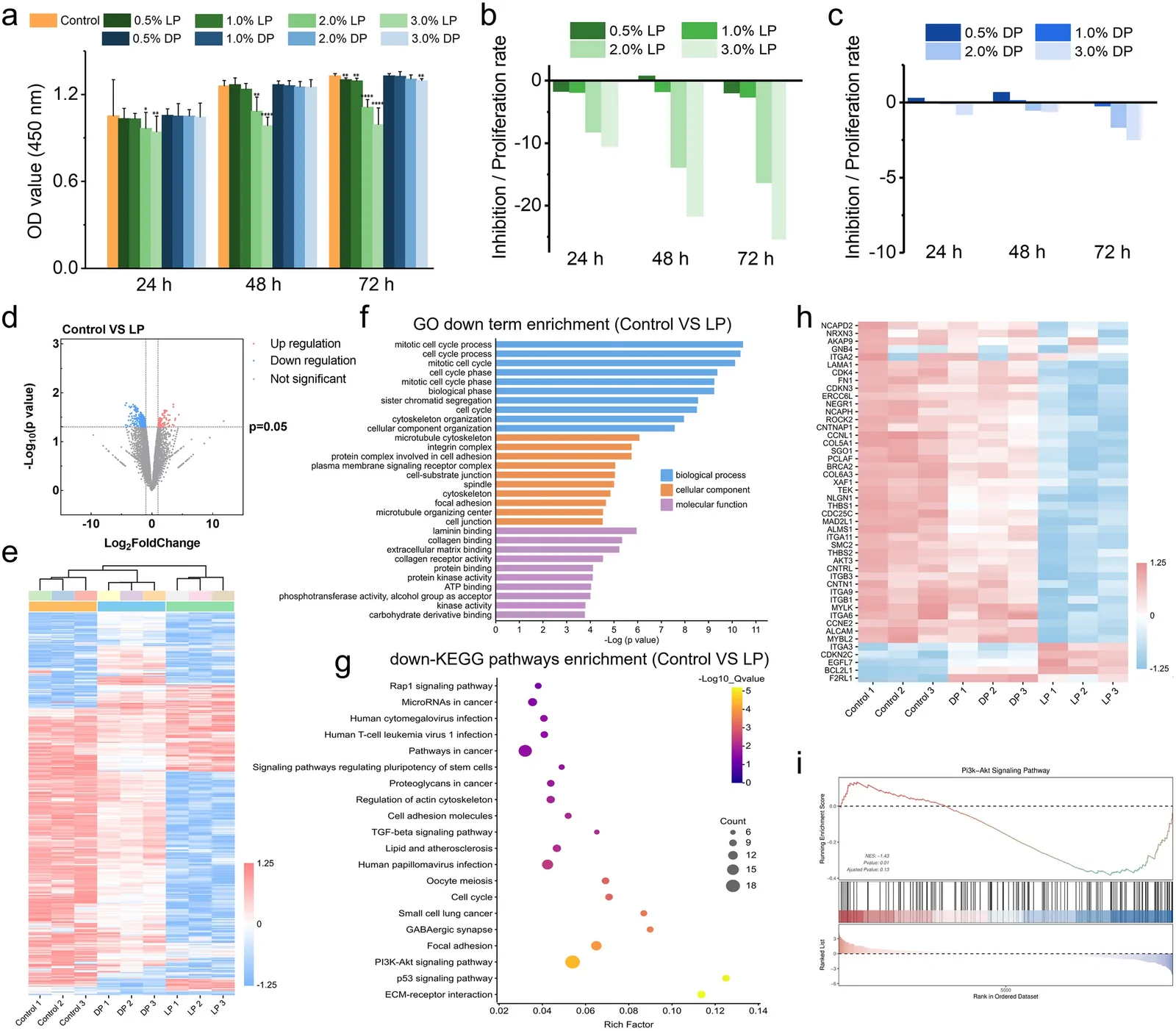
RNA-seq analysis of L/DP on HSFs. **a** Absorbance value of HSFs at 450 nm treated with different concentrations of L/DP for 24, 48, and 72 h (*n* = 3 independent samples). Corresponding inhibition or/and proliferation rate of different concentrations of **b** LP and **c** DP. **d** Volcano plot for differentially expressed genes between control and LP groups (*n* = 3 independent samples). The blue plots represent downregulated genes, and red plots indicate upregulated genes. **e** Heatmap of differentially expressed genes among control, DP, and LP groups. **f** Significantly enriched BP, CC, and MF of GO terms between control and LP groups. **g** The most significantly enriched KEGG signaling pathways between control and LP groups. The data in **f**, **g** are analyzed by hypergeometric distribution. The P values are adjusted using the Benjamini–Hochberg method. **h** Heatmap analysis of differentially expressed genes involved in integrin complex, cell cycle, cell adhesion, ECM construction, and the PI3K-Akt signaling pathway between control and LP groups. **i** GSEA of the “PI3K-Akt signaling pathway” with the GO-BP database between control and LP groups. Data are expressed as the mean ± SD. Differences among the groups were examined with t test. *p < 0.05, **p < 0.01, ***p < 0.001, ****p < 0.0001, and ns (not significant versus the indicated group)

Hypertrophic scar fibroblasts (HSFs), the principal functional cell type in HS, were used to investigate the biological effects of L/DP. LP showed an inhibitory effect on cell proliferation and presented a tendency toward concentration-dependent inhibition over time (Fig. 3a, b). In contrast, the inhibitory effect of DP on HSFs had no significant difference at all concentrations after 24, 48, and 72 h (Fig. 3c). A concentration of 3% for both LP and DP was therefore selected for subsequent experiments, as it produced the most pronounced effects on HSFs. To evaluate the selectivity of LP, we assessed its cytotoxicity on normal skin fibroblasts (NSFs). Notably, LP treatment resulted in minimal changes to NSF viability at concentrations that effectively suppressed HSFs (Fig. S14), indicating a favorable therapeutic window and high selectivity for pathological scar tissue. RNA sequencing (RNA-seq) was then performed. Volcano plots revealed 104 upregulated and 329 downregulated genes in Control vs LP, versus only 36 upregulated and 30 downregulated genes in control vs DP (Figs. 3d and S15). These differentially expressed genes (DEGs) were visualized by heatmaps (Fig. 3e). Gene Ontology (GO) terms and Kyoto Encyclopedia of Genes and Genomes (KEGG) pathways were employed to analyze the DEGs. GO analysis showed that downregulated DEGs in control vs LP were enriched in integrin complex, cell cycle, cytoskeleton organization, cell adhesion, focal adhesion, and extracellular matrix binding (Fig. 3f). Consistently, the top enriched downregulated KEGG pathways indicated reduced cell adhesion and proliferation (cell adhesion molecules, focal adhesion, PI3K-Akt signaling), and matrix formation (ECM–receptor interaction, TGF-β signaling) after LP treatment (Fig. 3g). Gene set enrichment analysis (GSEA) further confirmed that LP negatively regulated gene sets associated with focal adhesion, ECM–receptor interaction, and the PI3K–AKT signaling pathway (Figs. 3i and S16). Key DEGs included downregulated ITGβ1, fibronectin 1 (FN1), cyclin-dependent kinase 4 (CDK4), collagen type V alpha 1 (COL5A1), and v-akt murine thymoma viral oncogene homolog 3 (AKT3), together with upregulation of cyclin-dependent kinase inhibitor 2C (CDKN2C) (Fig. 3h). Collectively, these RNA-seq data suggest that LP reduces ITGβ1 expression and thereby inhibits abnormal HSF growth by modulating focal adhesion, PI3K–AKT, and TGF-β signaling pathways.

**Fig. 4:**
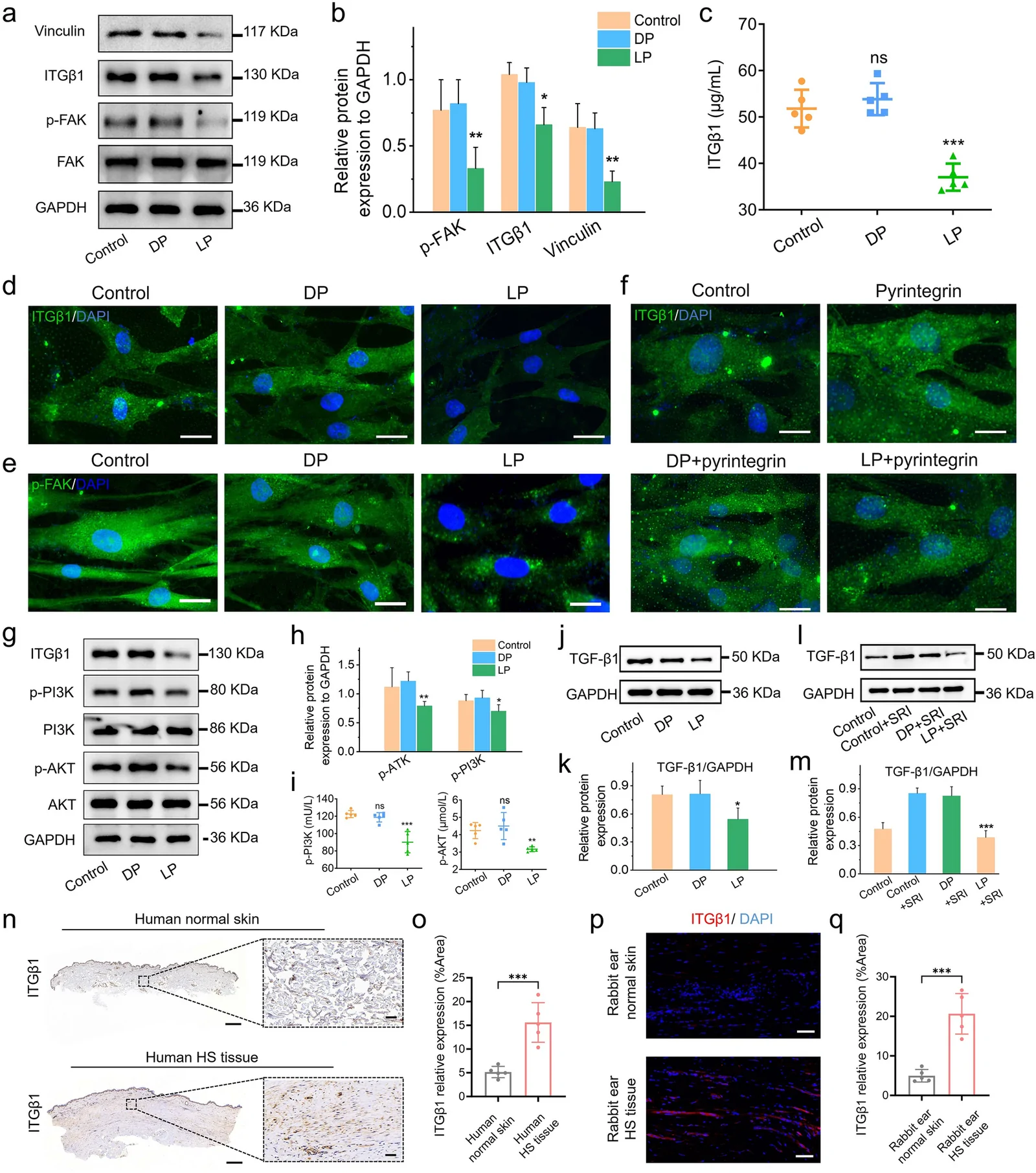
L/DP influences ITGβ1 and integrin-mediated signaling pathway on HSFs. **a** Representative immunoblots showing expression of vinculin, ITGβ1, p-FAK, and FAK on HSFs after varied treatments. **b** Quantitative analyses of the protein expression levels of Vinculin, ITGβ1, and p-FAK (*n* = 3 independent samples). **c** ELISA experiment results of ITGβ1 in the control, DP, and LP groups (*n* = 3 independent samples). Representative immunofluorescence images of **d** ITGβ1 (green) and DAPI (blue) and **e** p-FAK (green) and DAPI (blue) on HSFs after varied treatments. Scale bar, 50 μm. **f** Representative immunofluorescence images of ITGβ1 (green) and DAPI (blue) on HSFs with different treatments. Scale bar, 50 μm. **g** Immunoblots showing expression of ITGβ1, p-PI3K, PI3K, p-AKT, and AKT on HSFs in different groups. **h** Quantitative analyses of the protein expression levels of p-PI3K and p-AKT in different groups (*n* = 3 independent samples). **i** ELISA experiment results of p-PI3K and p-AKT in the control, DP, and LP groups (*n* = 3 independent samples). **j**, **l** Immunoblot images of TGF-β1 with various treatments. **k**, **m** The relative protein expression of TGF-β1 after various treatments (*n* = 3 independent samples). **n** Representative immunohistochemistry staining of ITGβ1 in human normal skin and human HS tissue. Scale bar, 1000 μm; Scale of the enlarged image, 50 μm. **o** Semiquantitative statistics of ITGβ1 in various group (*n* = 5). **p** Representative immunofluorescence images of ITGβ1 (red) and DAPI (blue) in rabbit ear normal skin and rabbit ear HS tissue. Scale bar, 50 μm. **q** Semiquantitative statistics of ITGβ1 in various group (*n* = 5). Data are expressed as the mean ± SD. Differences among the groups were examined with one-way ANOVA and *t* test. *p < 0.05, **p < 0.01, ***p < 0.001, ****p < 0.0001, and ns (not significant versus the indicated group)

Differential expression of selected genes was further validated at the protein level by Western blotting (WB). The protein expression level of ITGβ1 was found to be downregulated after LP treatment, while DP treatment resulted in elevated levels of ITGβ1, which were also confirmed by ELISA experiments (Fig. 4a–c). Immunofluorescence staining showed reduced ITGβ1 fluorescence intensity in LP-treated cells compared with control and DP-treated cells (Figs. 4d and S17). Treatment with pyrintegrin, a commonly used ITGβ1 agonist, abrogated the suppressive effect of LP on ITGβ1 levels (Figs. 4f and S18). Consistent with ITGβ1 downregulation, LP also markedly reduced phosphorylation of the downstream effector focal adhesion kinase (p-FAK) (Figs. 4e and S19). This decrease in p-FAK was further confirmed by immunofluorescence staining and ELISA (Figs. S20 and S21). Moreover, vinculin, a structural component of focal adhesion complexes, was also downregulated after LP treatment.

One of the major pathways downregulated by LP in the RNA-seq analysis was the PI3K–AKT signaling pathway, which operates downstream of integrin/FAK activation [49]. In the control and DP groups, ITGβ1, AKT, and PI3K were markedly activated, as evidenced by increased levels of ITGβ1, p-PI3K, and p-AKT in WB analyses (Fig. 4g). By contrast, LP treatment decreased the expression of ITGβ1, p-FAK, and p-AKT (Figs. 4h and S22), and this decreasing trend was further corroborated by ELISA (Figs. 4i and S23). Notably, these changes were not observed in the presence of pyrintegrin (Fig. S24). To validate the RNA-seq findings, the expression levels of key DEGs (ITGβ1, FN1, and AKT3) were also quantified by qRT-PCR. The qPCR results showed strong concordance with the transcriptomic data, confirming the downregulation of adhesion and survival pathways by LP treatment (Fig. S25). Together, these results demonstrate that LP downregulates ITGβ1 and inhibits the ITGβ1-mediated FAK/PI3K/AKT signaling cascade. In addition, KEGG pathway analysis identified enrichment of TGF-β signaling. A growing body of evidence indicates that integrins play a key role in latent TGF-β activation [50, 51]. WB experiments showed reduced TGF-β1 expression after LP treatment compared with the control and DP groups (Fig. 4j, k). Addition of SRI-011381, a TGF-β1 agonist, increased TGF-β1 expression, which was subsequently suppressed by LP treatment (Fig. 4l, m). These findings indicate that LP modulates ITGβ1 overexpression by reshaping the local microenvironment and blocking the ITGβ1/FAK/PI3K/AKT pathway. LP also exerts an inhibitory effect on TGF-β1, providing a potentially more effective intervention for HS.

Skin tissue samples from patients clinically diagnosed with HS (abbreviated as human HS tissue) were further examined for ITGβ1 expression. Healthy adjacent skin tissue samples (abbreviated as human normal skin) served as controls. Immunohistochemistry results showed that ITGβ1 was highly expressed in human HS tissue, with negligible levels detected in human normal skin (Fig. 4n). Statistical analysis demonstrated that the expression of ITGβ1 in human HS tissue was at least three times greater than that in normal skin (Fig. 4o). Immunofluorescence results corroborated the immunohistochemistry findings, revealing that semiquantitative ITGβ1 expression in human HS tissue was approximately six times higher than in human normal skin (Fig. S26). Hematoxylin and eosin (H&E) staining images illustrated a significant increase in subcutaneous thickness in human HS tissue, highlighting the typical pathological characteristics of HS [52]. Further, Masson and Sirius red staining revealed substantial dense and excessive collagen deposition in ITGβ1-overexpressing tissue (Fig. S27). The expression of ITGβ1 was then assessed in a rabbit ear HS model since it closely resembles human HS. Immunohistochemical and immunofluorescence staining analysis verified that rabbit ear HS tissue displayed higher ITGβ1 levels than rabbit ear normal skin (Figs. 4p and S28). About four times the amount of ITGβ1 expression was detected in rabbit ear HS tissue compared to rabbit ear normal skin (Fig. 4q). Similar pathogenic features were observed in rabbit ear HS tissue (Fig. S29). These results collectively indicated that ITGβ1 was highly expressed in human and rabbit HS tissues, underscoring the significance of regulating ITGβ1 expression in HS treatment.

### L/DP Modulates HSFs Behaviors via ITGβ1-Mediated Mechanisms

**Fig. 5:**
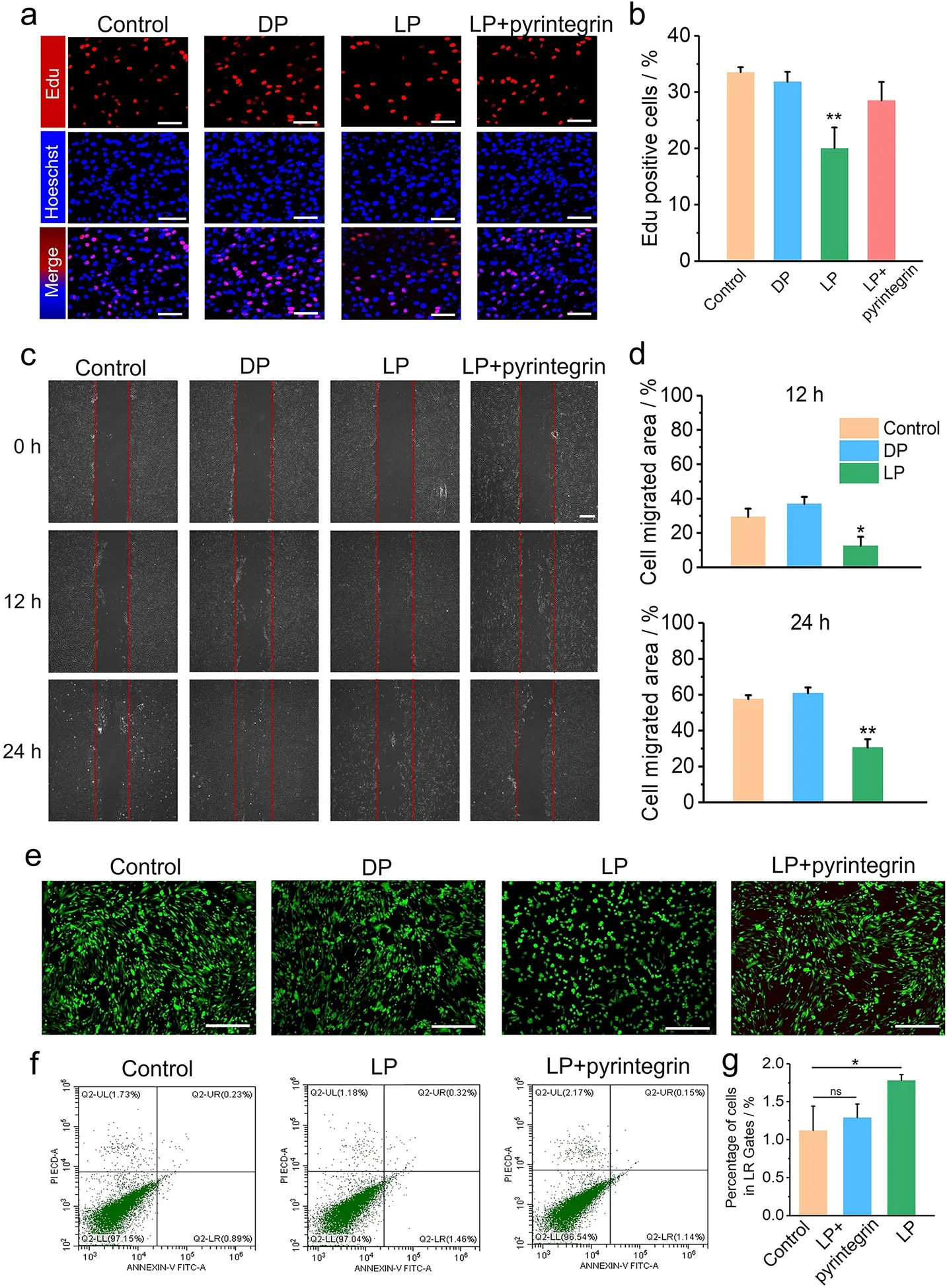

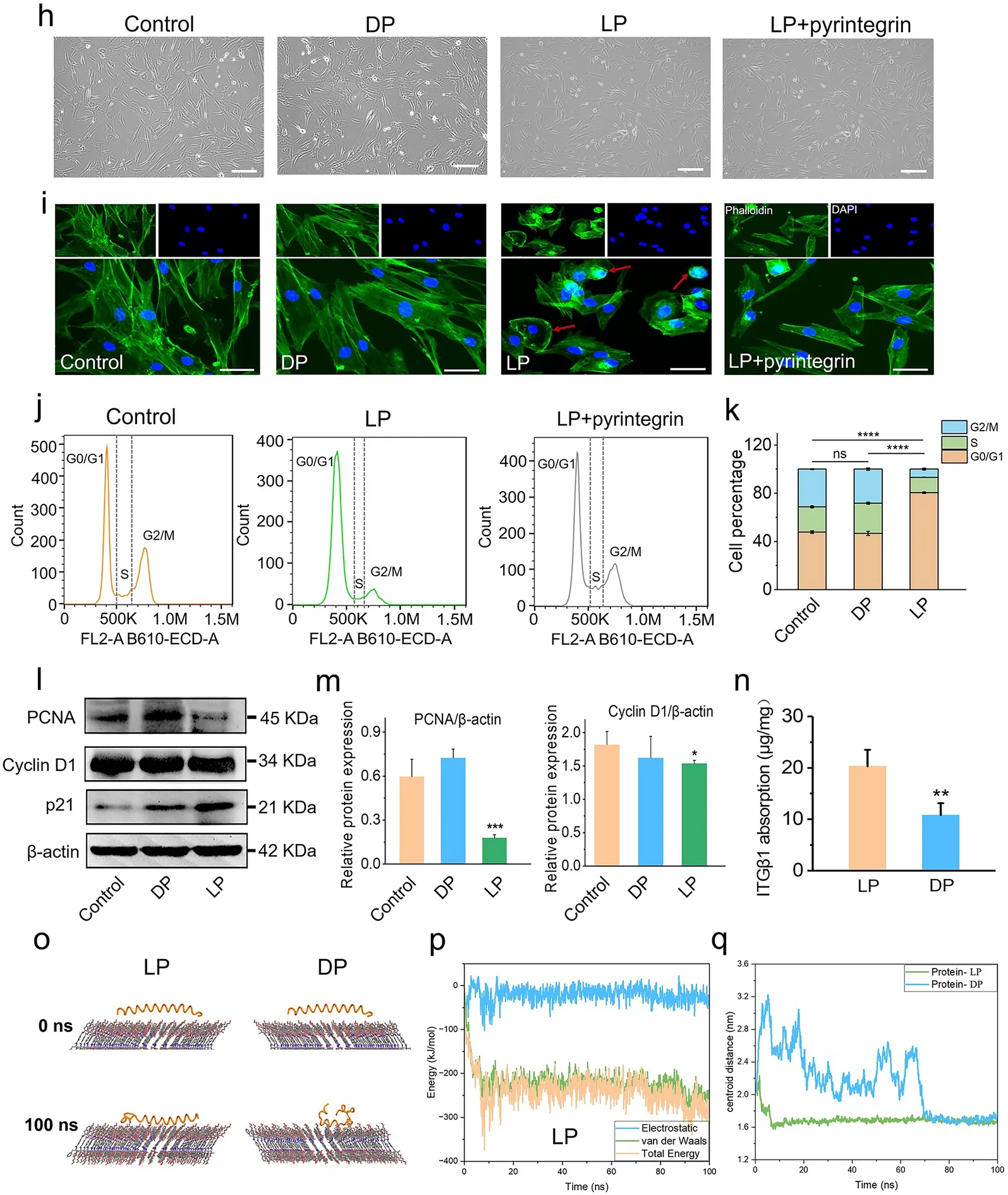
Inhibited excessive proliferation and migration of HSFs by LP via ITGβ1-mediated manner. **a** Representative images of Edu staining of HSFs after different treatments. Scale bar, 250 μm. **b** Corresponding percentage of Edu-positive cells after various treatments (*n* = 3 independent samples). **c** Representative photographs of cell migration with different treatments. Scale bar, 200 μm. **d** Corresponding percentage of cell-migrated area for 12 and 24 h (*n* = 3 independent samples). **e** Representative fluorescence images of a live/dead assay of HSFs after various treatments. Scale bar, 500 μm. **f** Flow cytometry results of HSFs after varied treatments. **g** Statistics for the percentage of cells in LR gates in **f** (*n* = 3 independent samples). **h** Representative bright-field microscopy images of HSFs after various treatments. Scale bar, 200 μm. **i** Fluorescence images of HSFs, blue (DAPI) for nuclei, and green (fluorescein isothiocyanate (FITC)-labeled phalloidin) for F-actin. Scale bar, 50 μm. **j** Cell cycle assay maps of HSFs after different treatments. **k** Corresponding percentage of G0/G1, S and G2/M phase cells (*n* = 3 independent samples). **l** Immunoblots showing expression of PCNA, cyclin D1, and p21 on HSFs after varied treatments. **m** Quantitative analyses of the protein expression levels of PCNA, cyclin D1, and p21 (*n* = 3 independent samples). **n** Protein adsorption of ITGβ1 on L/DP. **o** Representative snapshots of the system at different times. **p** Interaction energy between the protein and the substrate changes over the simulation time period. **q** Change of the centroid distance between the protein and the substrate over time during the simulation period. Data are expressed as the mean ± SD, *n* = 3 per group. Differences among the groups were examined with one-way ANOVA and t test. *p < 0.05, **p < 0.01, ***p < 0.001, ****p < 0.0001, and ns (not significant versus the indicated group)

ITGβ1 was overexpressed in HSFs compared with normal fibroblasts. HSFs with elevated ITGβ1 displayed higher proliferative capacity than normal fibroblasts, whereas treatment with an anti-ITGβ1 antibody attenuated aberrant cell growth, indicating that ITGβ1 contributes to HSF proliferation (Fig. S30). We next examined ITGβ1-dependent cellular behaviors regulated by L/DP. EdU incorporation assays showed that LP reduced HSF proliferation to ~ 20% EdU⁺ (EdU-positive) cells, whereas DP had no significant effect (Fig. 5a, b). The cell scratch test demonstrated faster migration of HSFs in the control and DP groups (Fig. 5c). LP inhibited cell migration by 17% (12 h) and 27% (24 h), respectively, while pyrintegrin treatment enhanced cell migration (Fig. 5d). Live/dead staining revealed abundant green fluorescence (viable cells) in the control and DP groups after 48 h, whereas LP treatment decreased green fluorescence with a modest increase in red fluorescence (dead cells) (Fig. 5e). The number of cells was further quantified by flow cytometry. No obvious statistical difference in apoptosis was visualized in the control and pyrintegrin treatment groups (Fig. 5f). However, the percentage of apoptotic cells slightly increased after LP treatment (Figs. 5g and S31). Morphological changes in HSFs were consistent with these alterations in proliferation and migration. In the control and DP groups, HSFs exhibited a spindle-shaped morphology with disordered orientation (Fig. 5h). In contrast, LP-treated cells showed reduced cell density and adopted a curled or rounded morphology, which was partially reversed by pyrintegrin (Fig. 5i).

Because LP had only a minor effect on apoptosis, we investigated whether it inhibited proliferation by altering the cell cycle. Based on PI fluorescence intensity, the G0/G1 phase was defined as cells with a DNA content of 2*n* (first peak), the S phase as cells with intermediate DNA content between 2 and 4n, and the G2/M phase as cells with a DNA content of 4*n*. Flow cytometry analysis revealed synchronization rates of the G0/G1 phase, S phase, and G2/M phase in the control group to be 47.81%, 20.89%, and 31.29%, respectively, after 24 h of cultivation (Fig. 5j, k). LP significantly increased the proportion of cells in the G0/G1 phase (from 47.81% to 80.44%) while decreasing the percentages in the S phase (from 20.89% to 12.78%) and G2/M phase (from 31.29% to 6.77%) compared to the control group. This indicated that LP could induce G0/G1 cycle arrest in HSFs. Pyrintegrin treatment conversely increased the proportion of cells in the active phases (S and G2/M) and decreased the percentage in the G0/G1 phase (Fig. S32). WB experiment was conducted to assess the expression of potential cell cycle-related regulatory proteins involved in G0/G1 arrest. The cyclin-dependent kinase inhibitor p21 and cyclin D1 are two AKT-regulated proteins that negatively (p21) and positively (cyclin D1) control cell cycle progression. It was observed that the protein expression of cyclin D1 decreased while p21 increased after LP treatment (Figs. 5l, S33, and S34) and also reduced proliferating cell nuclear antigen (PCNA), a marker of proliferation (Fig. 5m). ELISA provided additional evidence for decreased cyclin D1 and increased p21 after LP treatment, whereas pyrintegrin partially reversed these changes (Fig. S35). Collectively, these findings indicate that LP suppresses HSF proliferation and migration predominantly through ITGβ1-mediated mechanisms involving cell cycle arrest rather than apoptosis.

To further confirm the stereoselective recognition of ITGβ1 proteins with chiral self-assembly nanofibers, we investigated the interactions between ITGβ1 and L/DP. From Fig. 5n, the adsorption capacity of ITGβ1 on LP was obviously higher than that on DP treatment for 2 h. To gain molecular-level insight into this chiral preference, we performed molecular dynamics (MD) simulations to examine the adsorption modes of ITGβ1 on L/DP. The simulations revealed that ITGβ1 preferentially adsorbed onto LP, attributable to the left-handed helical architecture of the LP fibers. At the beginning of the simulations, the protein was placed at the same position above the surfaces of both chiral substrates (Fig. 5o). After 100 ns, ITGβ1 lay flat on the LP surface, forming an extended contact interface, whereas on the DP surface, the protein adopted a bent conformation with reduced contact area, consistent with weaker adsorption. We next quantified the interaction energies between ITGβ1 and each substrate over time (Figs. 5p and S36). The binding was dominated by van der Waals interactions, with relatively minor electrostatic contributions. Notably, adsorption of ITGβ1 on the LP surface reached equilibrium rapidly, and the adsorption energy was substantially more favorable than that for DP. Finally, we calculated the time-dependent distance between the protein center of mass and the centroids of the two substrates (Fig. 5q). ITGβ1 rapidly approached and remained stably associated with the LP surface, whereas on DP, it underwent cycles of desorption and re-adsorption and maintained a larger average separation, in agreement with the interaction energy profiles and structural snapshots. Interestingly, while LP significantly downregulated ITGβ1, the DP resulted in a modest upregulation of ITGβ1 expression. This phenomenon could be explained by the “Compensatory Adhesion Hypothesis.” Our MD simulations reveal that DP binds ITGβ1 weakly and unstably due to chiral mismatch. In response to this mechanically unstable interface, fibroblasts likely upregulate integrin expression to compensate and maintain focal adhesion. This pro-adhesive potential of the DP underscores the critical importance of enantiomeric purity in the design of supramolecular therapeutics, confirming that only the LP nanostructure confers the specific safety and efficacy profile required for HS therapy. Together, these results support the conclusion that left-handed LP nanofibers enhance stereoselective interactions with ITGβ1, thereby contributing to G0/G1 cell cycle arrest in HSFs.

### Therapeutic Effects of MNs in Rabbit Ear HS Models

**Fig. 6:**
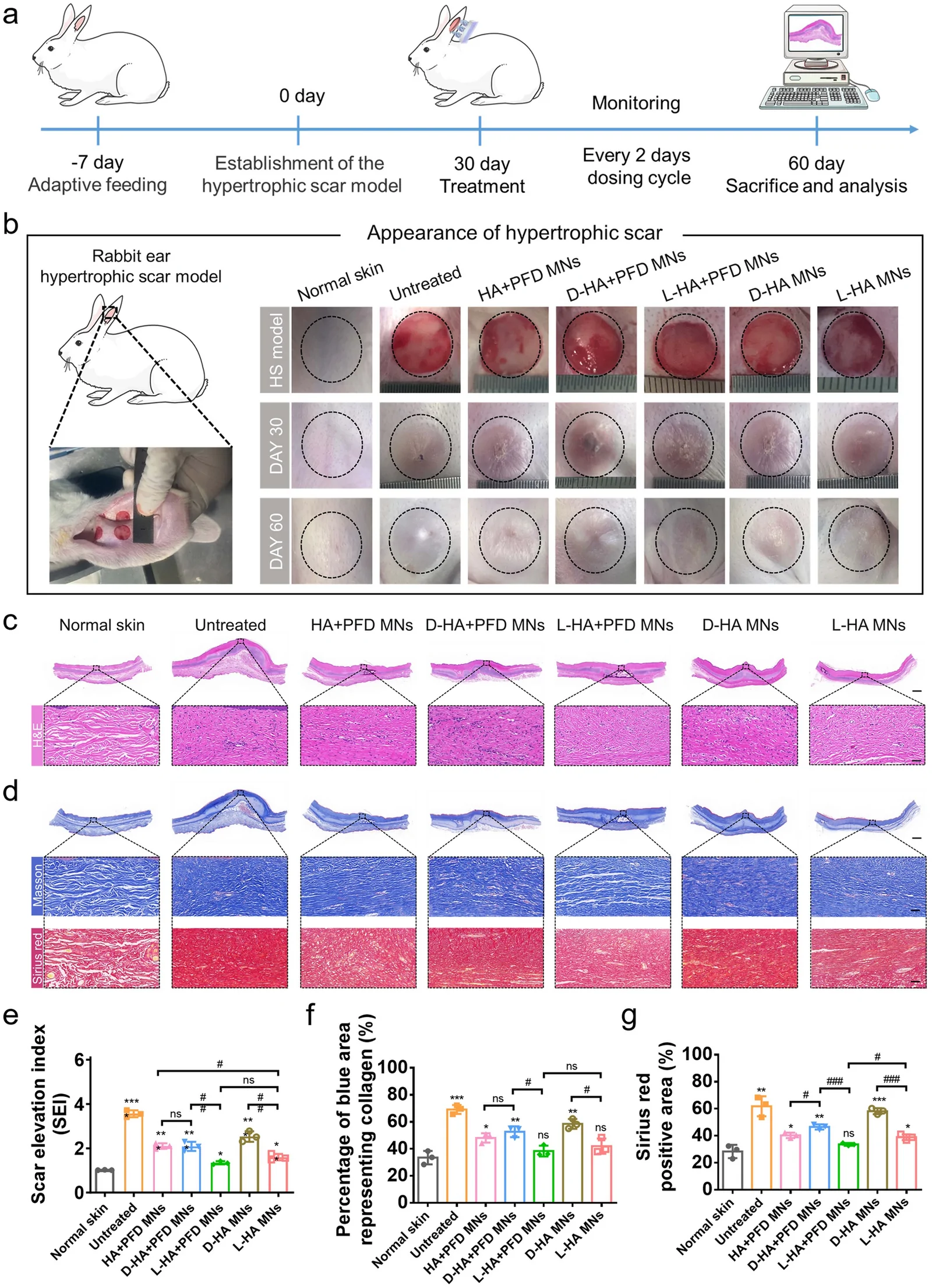
Evaluation of therapeutic effect on rabbit ear HS models under different interventions. **a** Experimental schematic of various MNs for HS therapy. **b** Photographs of scars on rabbit ears after various treatments at different times. **c** Representative H&E staining of HS on day 60. Scale bar, 1000 μm in H&E staining images (top) and 50 μm in enlarged images. **d** Representative Masson staining and Sirius red staining images of HS on day 60. Scale bar, 1000 μm in Masson staining images (top), 50 μm in Masson staining images (enlarged), and Sirius red staining images (enlarged). Quantitative analysis of **e** SEI, **f** percentage of collagen, and** g** percentage of Sirius red positive area of HS (*n* = 3 independent samples). Data are expressed as the mean ± SD, *n* = 5 per group. Differences among the groups were examined with one-way ANOVA and t test. *p < 0.05, **p < 0.01, ***p < 0.001, ****p < 0.0001, and ns (not significant versus the indicated group)

Rabbit ear HS models were established 30 days post-wounding (Fig. 6a). Treatments included PBS (normal), untreated, HA + PFD MNs (positive control), D-HA MNs, and L-HA MNs. In the untreated group, HS continued to progress up to day 60, with persistently raised scar surfaces (Fig. 6b). D-HA MNs induced only mild scar regression, whereas L-HA MNs and HA + PFD MNs produced markedly flatter scar tissue that approached the appearance of normal skin by 30 days of treatment (Fig. S37). Hematoxylin and eosin (H&E) staining revealed pronounced epidermal and dermal hyperplasia accompanied by dense infiltration of proliferative fibroblasts in the untreated group (Fig. 6c). These histopathological features were not substantially alleviated by D-HA MNs. By contrast, L-HA MNs and HA + PFD MNs markedly improved scar architecture, yielding a thinner, flatter epidermis. This effect was particularly pronounced after L-HA MNs treatment, which nearly restored the skin to the normal level. This improvement was quantitatively assessed by the scar evaluation index (SEI).

This improvement was quantitatively assessed using SEI. The SEI in the untreated group was 3.5-fold higher than that in the normal skin group (Fig. 6e). Following treatment with HA + PFD MNs, D-HA MNs, and L-HA MNs, SEI values decreased to 2.10, 2.53, and 1.60, respectively. Masson staining demonstrated excessive, disorganized collagen fiber deposition in the untreated group, with a blue-stained area (collagen) of 69.15% (Fig. 6d). After treatment with L-HA MNs and HA + PFD MNs, collagen density was significantly reduced and fibers appeared more regularly aligned, with blue-stained areas of 48.16% and 42.04%, respectively (Fig. 6f). Consistent results were obtained with Sirius red staining, where the Sirius red–positive area decreased from a maximum of 61.79% in the untreated group to a minimum of 38.91% after L-HA MNs treatment. Furthermore, among all interventions, L-HA MNs achieved the greatest reduction in scar thickness, with an observed 23% improvement in scar thickness (Table 1), suggesting its potential as a potent anti-scarring therapy. Together, these findings demonstrate that L-HA MNs effectively inhibit fibroblast overproliferation and collagen deposition, providing the most pronounced therapeutic benefit in this HS model.

**Table 1:** Improvement in scar thickness after various intervention methods

Intervention method	Degree of improvement (SEI)	References
Free quercetin	14%	[50]
Photodynamic therapy	10.7%	[51]
Laser therapy	− 10%	[52]
5-fluorouracil	13.3%	[53]
Triamcinolone acetonide-bilayer dissolving microneedle	− 6%	[26]
LP biomaterial	23%	This study

The degree of improvement was obtained by comparing the SEI values between the treatment group and the HS model group

**Fig. 7:**
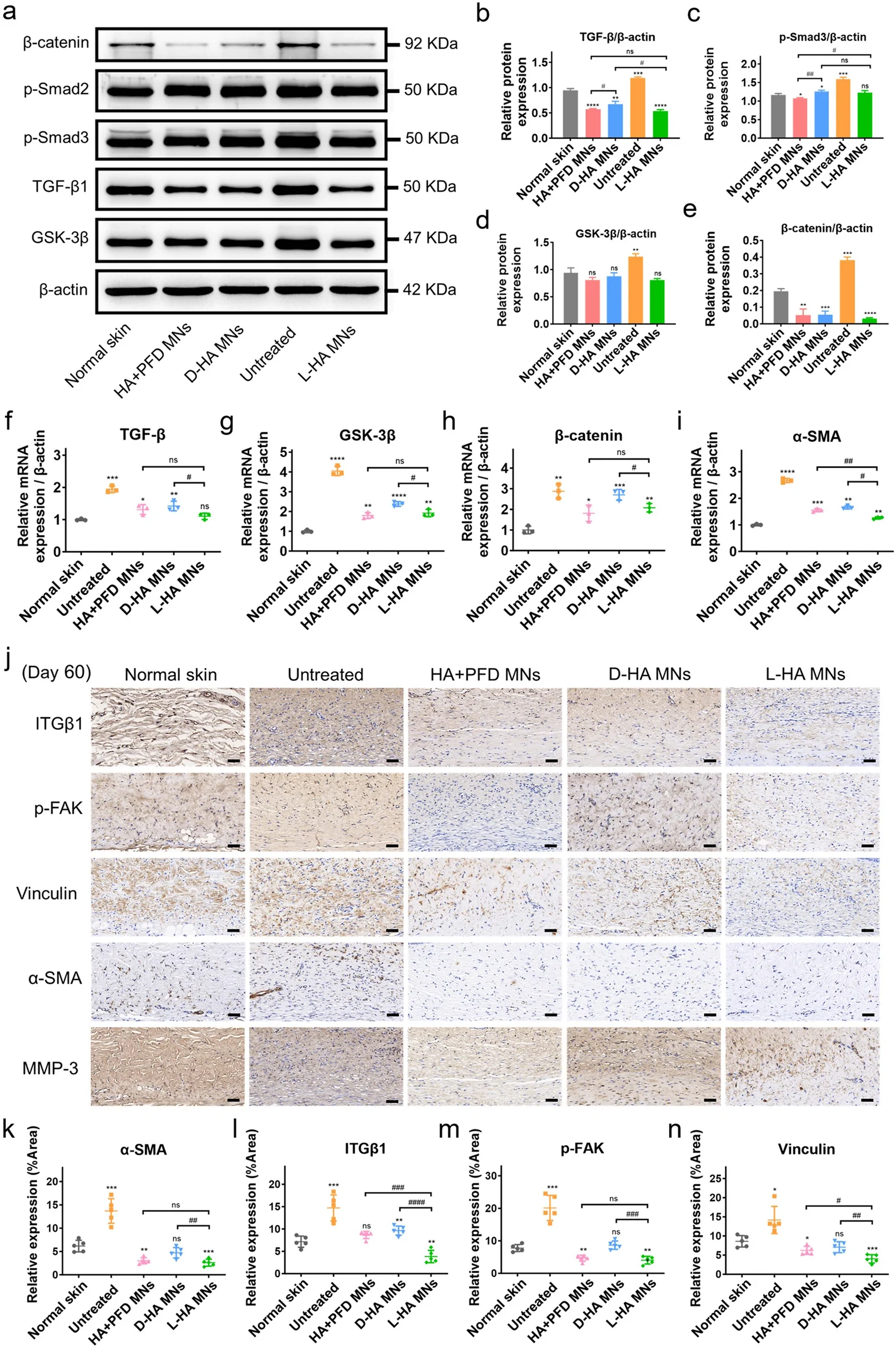
MNs regulating of the pathological microenvironment and influencing the integrin β1 and TGF-β/Wnt-mediated signaling pathways for HS intervention. **a** Immunoblots showing expression of β-catenin, p-Smad2, p-Smad3, TGF-β1, and GSK-3β on rabbit ear HS after varied treatments. Quantitative analyses of the protein expression levels of **b** TGF-β1, **c** p-Smad3, **d** GSK-3β, and** e** β-catenin in different groups (*n* = 3 independent samples). Quantitative analyses of the relative mRNA expression levels of **f** TGF-β1, **g** GSK-3β,** h** β-catenin, and **i** α-SMA after different treatments. **j** Representative immunohistochemistry staining of ITGβ1, p-FAK, vinculin, α-SMA, and MMP-3 on day 60 in various groups. Scale bar, 50 μm. Semiquantitative statistics of** k** ITGβ1, **l** p-FAK, **m** vinculin, and** n** α-SMA in different groups. Data are expressed as the mean ± SD. Differences among the groups were examined with one-way ANOVA and t test. *p < 0.05, **p < 0.01, ***p < 0.001, ****p < 0.0001, and ns (not significant versus the indicated group)

WB was used to assess protein expression after the different treatments (Fig. S38). The expression level of TGF-β1, a key factor in scar formation, was significantly higher in the untreated group than in the treated groups (Fig. 7a). The relative protein expression of TGF-β1 in each MNs group was significantly reduced, with the L-HA MNs and HA + PFD MNs groups showing particularly notable decreases (Fig. 7b). Similarly, the mRNA expression level of TGF-β1 was significantly elevated in the untreated group but substantially decreased after MNs treatment (Fig. 7f). Smad2 and Smad3 are intracellular substrates of the TGF-β1 receptor that become phosphorylated upon TGF-β1 activation and participate in the transcription of key genes involved in HS formation [26, 53, 54]. The levels of p-Smad2 and p-Smad3 were higher in the untreated group, whereas they were significantly attenuated after various MNs treatments (Figs. 7c and S39). We next examined additional proteins involved in fibroblast activation and scar formation. β-Catenin and glycogen synthase kinase-3β (GSK-3β) were highly expressed in the untreated group, whereas their protein levels were markedly reduced after MN treatment (Fig. 7d, e). In the L-HA MNs group, GSK-3β protein expression was 0.86-fold that of normal skin, and β-catenin expression was reduced to 0.16-fold of normal skin. Similar trends were observed at the mRNA level (Fig. 7g, h). Furthermore, the untreated group exhibited a 2.6-fold increase in α-smooth muscle actin (α-SMA), a marker of activated fibroblasts, at the mRNA level compared with the normal skin group [55]. In contrast, relative α-SMA expression decreased to 1.23-, 1.65-, and 1.55-fold of normal skin after treatment with L-HA MNs, D-HA MNs, and HA + PFD MNs, respectively (Fig. 7i). Immunofluorescence staining further confirmed widespread and intense α-SMA distribution throughout the dermis and epidermis in the untreated group (Figs. 7j and S40), whereas α-SMA expression was markedly reduced after MN treatment. Semiquantitative analysis showed that α-SMA expression in the untreated group was approximately 2.2-fold higher than in normal skin, with the lowest levels observed in the L-HA MNs group (Fig. 7k). Consistent with the α-SMA findings, matrix metalloproteinase-3 (MMP-3) expression decreased in parallel with HS improvement after L-HA MNs treatment and was lower than in the other groups (Fig. S41). Collectively, these data indicate that the modulation of TGF-β1 signaling and fibroblast activation-related proteins is critical for the therapeutic effects of MN-based treatment in HS.

Finally, integrin-related proteins were examined by immunofluorescence staining. ITGβ1 was markedly overexpressed in the untreated group but was significantly reduced after MN treatment (Fig. 7l). Notably, ITGβ1 expression in the HA + PFD MNs group was lower than in the D-HA MNs group, whereas ITGβ1 in the L-HA MNs group was almost undetectable. Additionally, the expression levels of p-FAK and vinculin were substantially elevated in the untreated group but declined after MN treatment (Fig. 7j). Quantitative analysis showed that the relative levels of p-FAK and vinculin decreased after MN treatment, with the greatest reduction observed in the L-HA MNs group (Fig. 7m, n). Collectively, these results demonstrate that the LP biomaterial could stereoselectively regulate ITGβ1 to suppress profibrotic signaling pathways (FAK/PI3K/AKT, TGF-β), inhibit HSF hyperactivity, and reduce scar thickness and collagen deposition. Unlike previous chiral biomaterials that primarily reported phenomenological differences in cell adhesion or proliferation, our study integrates (1) molecular dynamics–guided analysis of chiral ITGβ1 adsorption, (2) receptor-specific validation of ITGβ1 clustering and downstream signaling, and (3) functional in vivo regeneration outcomes. Overall, these results establish a direct mechanistic link between the chiral nanostructures and integrin-mediated tissue repair. This chirality-dependent strategy therefore provides a drug-free and highly effective approach for HS therapy.

## Conclusions

In summary, we developed a biomimetic chiral supramolecular material (L/DP) via chirality-driven self-assembly, specifically engineered to modulate the pathological microenvironment of HS. By mimicking the structural and optical features of the native ECM, L/DP formed well-defined chiral nanostructures with distinct chiroptical activity, thereby enabling stereoselective engagement of integrin β1 (ITGβ1) in scar tissue and attenuation of downstream profibrotic signaling. Mechanistically, LP inhibited fibroblast hyperproliferation by downregulating ITGβ1 expression by 72% and suppressing ITGβ1-mediated FAK/PI3K/AKT signaling and TGF-β1 activation. In vivo, LP reduced scar thickness by 54%, collagen accumulation by 39%, and α-SMA expression by 45%, outperforming conventional therapies (corticosteroids, laser therapy, photodynamic therapy) by 23% in scar thickness improvement. Overall, this chirality-dependent biomaterial provides a robust and translational strategy for HS therapy, addressing longstanding challenges in clinical scar management.

## Supplementary Information

Below is the link to the electronic supplementary material.

Supplementary file1 (DOCX 12447 KB)
