# Supplementary material for: Chirality-Dependent Supramolecular Biomaterials Remodeling of Scar Microenvironment via Integrin-Mediated Regulation for Hypertrophic Scars Therapy
Source: Nanomicro Lett. 2026 Apr 24;18:343. doi: 10.1007/s40820-026-02180-1 (PMC13109467; doi:10.1007/s40820-026-02180-1)
Supplement: Supplementary file 1 — Supplementary file1 (DOCX 12447 KB) [file 40820_2026_2180_MOESM1_ESM.docx]

Supporting Information for

**Chirality-Dependent Supramolecular Biomaterials Remodeling of Scar Microenvironment via Integrin-Mediated Regulation for Hypertrophic Scars Therapy**

Xueqian Wang^1, 2, +^, Chengyao Han^2, +^, Hongrui Shan^3, +^, Jinjin Li^4^, Beibei Wu^1^, Yixin Zhang^2,^ *, Ke Li^2,^ *, Chuanliang Feng^1,^ *

^1^ State Key Lab of Metal Matrix Composites, Shanghai Key Laboratory for Molecular Engineering of Chiral Drugs, School of Materials Science and Engineering, Shanghai Jiao Tong University, Shanghai 200240, P. R. China

^2^ Department of Plastic and Reconstructive Surgery, Shanghai Ninth People’s Hospital, Shanghai Jiao Tong University School of Medicine, Shanghai, P. R. China

^3^ Research Center of Precision Sensing and Control, Institute of Automation, Chinese Academy of Sciences, Beijing 100190, P. R. China

^4^ National Key Laboratory of Advanced Micro and Nano Manufacture Technology, Shanghai Jiao Tong University, Shanghai, 200240, P. R. China

^+^ Xueqian Wang, Chengyao Han, and Hongrui Shan contributed equally to this work.

*Corresponding authors. E-mail: [clfeng@sjtu.edu.cn](mailto:clfeng@sjtu.edu.cn) (Chuanliang Feng); [zhangyixin6688@hotmail.com](mailto:zhangyixin6688@hotmail.com) (Yixin Zhang); [18817821624@163.com](mailto:18817821624@163.com) (Ke Li )

**S1 Supplementary Materials and Methods**

**S1.1 Materials**

Pirfenidone and hyaluronic acid (HA, 10kDa) were bought from Aladdin Chemistry (Shanghai) Co., Ltd.; Terephthaloyl Chloride, D/L-Phenylalanine Methyl Ester Hydrochloride, were bought from Sigma-Aldrich Company; Ethanol (C_2_H_5_OH), Methanol (CH₃OH), Diethylene Glycol (C_4_H_10_O_3_), Dichloromethane (CH_2_Cl_2_), Triethylamine (C_6_H_15_N) and Ammonia were purchased from Macklin Biochemical (Shanghai) Co., Ltd. All aqueous solutions were prepared using ultrapure water (18 MU) from a Milli-Q system (Millipore). Calcein-AM/propidium iodide (PI), Hoechst 33258, Cell Counting Kit-8 (CCK-8, Dojindo), Cell Cycle Analysis Kit (KeyGEN), AnnexinV/PI Apoptosis Kit (KeyGEN), EDU Kit (Invitrogen), Triton X-100, bovine serum albumin (BSA), and 4% paraformaldehyde and were acquired from Shanghai Shaoxin Biotechnology Co., Ltd.; Fetal bovine serum (FBS), Trypsin (0.25%), Dulbecco’s Modified Eagle Medium (DMEM), and normal saline were purchased from Thermo Fisher Scientific (China) Co., Ltd.; Phalloidin-iFluor 488 conjugate was bought from ATT Bioquest; Primary hypertrophic scar fibroblasts (HSFs) were isolated from human pathological hypertrophic scar tissues, provided by the Department of Plastic and Reconstructive Surgery in the Shanghai Ninth People’s Hospital, Shanghai, China. All of the chemicals were used as received without further purification.

**S1.2 Synthesis of D/LP**

Based on a previous synthetic approach, L/D-phenylalanine chiral gelator (L/DPFEG) was synthesized.^1^ Terephthaloyl chloride (2.6 g, 13.0 mmol) was dissolved in DCM (20 mL) and slowly added to a mixture of 100 mL triethylamine (8.0 mL, 58.4 mmol) and L-phenylalanine methyl ester hydrochloride (6.0 g, 26.2 mmol) while being magnetically stirred. The mixture was agitated overnight to remove the excess solvent, and then concentrated using rotavapor. Following the ethanol-based dissolution of the remaining component in the flask, filtering, and drying, the insoluble materials p-Ph(L-Phe-OMe)_2_ were produced. The intermediate product (5.1 g, 10.5 mmol, 85%) was dissolved in methanol and NaOH aqueous solution (15 mL) was added while being stirred for 24 hours to produce a transparent solution. In order to produce gel precipitation, the pH of the solution was then changed using HCl (3.0 M). The gel was next filtered, several times washed with DI water, and vacuum dried to get the p-Ph(L-Phe-OH)_2_ (4.6 g, 9.9 mmol, 90%). Overall yield of LPF: 76.5%. Similarly, DPF was obtained as a white solid after freeze-drying (4.2 g, 82%).

After reacting at 130 °C for 3.5 hours, the cleaned solution was added to the frozen water, producing gel precipitation. After several filters and rinses with DI water, the sediment was finally baked to produce LPFEG gelator (4.2 g, 6.6 mmol, 91%), which was obtained. In the same way, DPFEG gelator (3.6 g, 5.6 mmol, 85%) was produced.

**S1.3 In vitro cell migration experiment**

The scratch assay was performed on the hypertrophic scar fibroblasts (HSFs) to assess cell migration. The relative Cell migration rate was calculated as following Equation (S1):

Cell migration rate = [(A0–An)/(A0)] × 100% (S1)

where A0 is the initial wound area (time = 0 h) and An is the wound area at each time point (24 h and 48 h). Data were presented as a mean with SD of three independent experiments.

**S1.4 In vivo histological assay**

For histological assays, scar tissues from rabbit ear were collected at day 30 and stained with Hematoxylin and Eosin (H&E). Based on H&E images, the scar elevation index (SEI) was quantified by Image J V. 1.52 and was calculated by the Equation (S2):

SEI = a / b (S2)

where a defines the maximum thickness of the scar tissue and brepresents the maximum thickness of the normal dermis surrounding the scar tissue. These values were determined by measuring the maximum thickness of the scar and normal tissues, respectively, from the top point of the epithelium to the surface of the cartilage.

**S2 Supplementary Tables**

**Table S1** Skin irritation test of HA MNs, L-HA MNs, and D-HA MNs in rabbit ear skin

| Group | Stimulus response | Time / h | | |
| --- | --- | --- | --- | --- |
|  |  | 1 | 24 | 72 |
| Control | erythema | 0 | 0 | 0 |
|  | edema | 0 | 0 | 0 |
| HA MNs | erythema | 0 | 0 | 0 |
|  | edema | 0 | 0 | 0 |
| L-HA MNs | erythema | 0 | 0 | 0 |
|  | edema | 0 | 0 | 0 |
| D-HA MNs | erythema | 0 | 0 | 0 |
|  | edema | 0 | 0 | 0 |

**Table S2** Western blotting assay

| Reagent | Company | Cat.ID |
| --- | --- | --- |
| RIPA Lysis Buffer | Bryotime | P0013B |
| PMSF（100mM） | Biosharp | BL507A |
| Phosphorylated Protease Inhibitor | Bryotime | P1081 |
| BCA protein assay kit | Bryotime | P0012 |
| SDS-PAGE loading buffer (5×) | Bryotime | P0015 |
| SDS-PAGE Gel Kit | Biosharp | BL508A |
| Protein Marker | YEASEN | 20350ES72 |
| TRIS | BIOFROXX | 1115GR500 |
| Glycine | BIOFROXX | 1275KG2P5 |
| SDS | BIOFROXX | 3250GR500 |
| PVDF membrane (0.45 μm)  PVDF membrane (0.22 μm)  BSA | Millipore  Millipore  Roche | IPVH00010  ISEQ00010  G5001 |
| TWEEN 20 | Solarbio | T8220 |
| ECL  Transfer buffer  Electrophoresis buffer  TBS buffer  PCNA | Biosharp  Boerfu  Boerfu  Boerfu  Proteintech | BL520A  BER0001  BER0002  BER0003  10205-2-AP |
| Cyclin D1 | Abcam | ab134175 |
| CDK4 | Abcam | ab199728 |
| p21 | Proteintech | 10355-1-AP |
| Intrgrinβ1 | Proteintech | 12594-1-AP |
| Vinculin | Proteintech | 66305-1-Ig |
| FAK  p-FAK  AKT  p-AKT  PI3K  p-PI3K  β-actin  GAPDH  β-catenin  p-Smad2  p-Smad3  TGF β1  GSK-3β | Affinity  Affinity  Proteintech  HUABIO  HUABIO  Affinity  Abclonal  HUABIO  CST  Bioss  Bioss  Proteintech  Bioss | AF6397  AF3398  60203-2-Ig  ET1612-73  ET1608-70  AF3241  AC026  ET1601-4  8480S  bs-3420R  bs-3425R  21898-1-AP  bs-0028R |
| HRP, Goat Anti-Rabbit IgG | Jackson | 111-035-003 |
| HRP, Rabbit Anti-Goat IgG | Jackson | 305-035-003 |
| HRP, Goat Anti-Mouse IgG | Jackson | 115-035-003 |
| HRP, Goat Anti-Rat IgG | Jackson | 112-035-003 |

**Table S3** Primers used for real-time PCR

| Target gene | Primer sequences (5′-3′) | |
| --- | --- | --- |
| β-actin | Forward  Reverse | CTGGAACGGTGAAGGTGACA  TCAAAGTCCTCGGCCACATT |
| GSK-3β | Forward  Reverse | TGTCCTTCTTCGTGGTGAGC  CATCTCGGACGTCAGGTAGC |
| TGF-β | Forward  Reverse | CGGAGAGCAGCCGGAAAAT  AAGCTGTGGATTGCTTTGCG |
| p-Smad3 | Forward  Reverse | GTGAGAAGGCGGTCAAGAGC  AGGGATTCACGCAAACCTCA |
| p-Smad2 | Forward  Reverse | GTGTGAGAAGGCCGTGAAGA  TCAGTCCCCAAATTTCAGAGCA |
| β-catenin | Forward  Reverse | ATGACTCGAGCTCAGAGGGT  GTTAGTGGGATGAGCGGCAT |

**S3 Supplementary Figures**

**
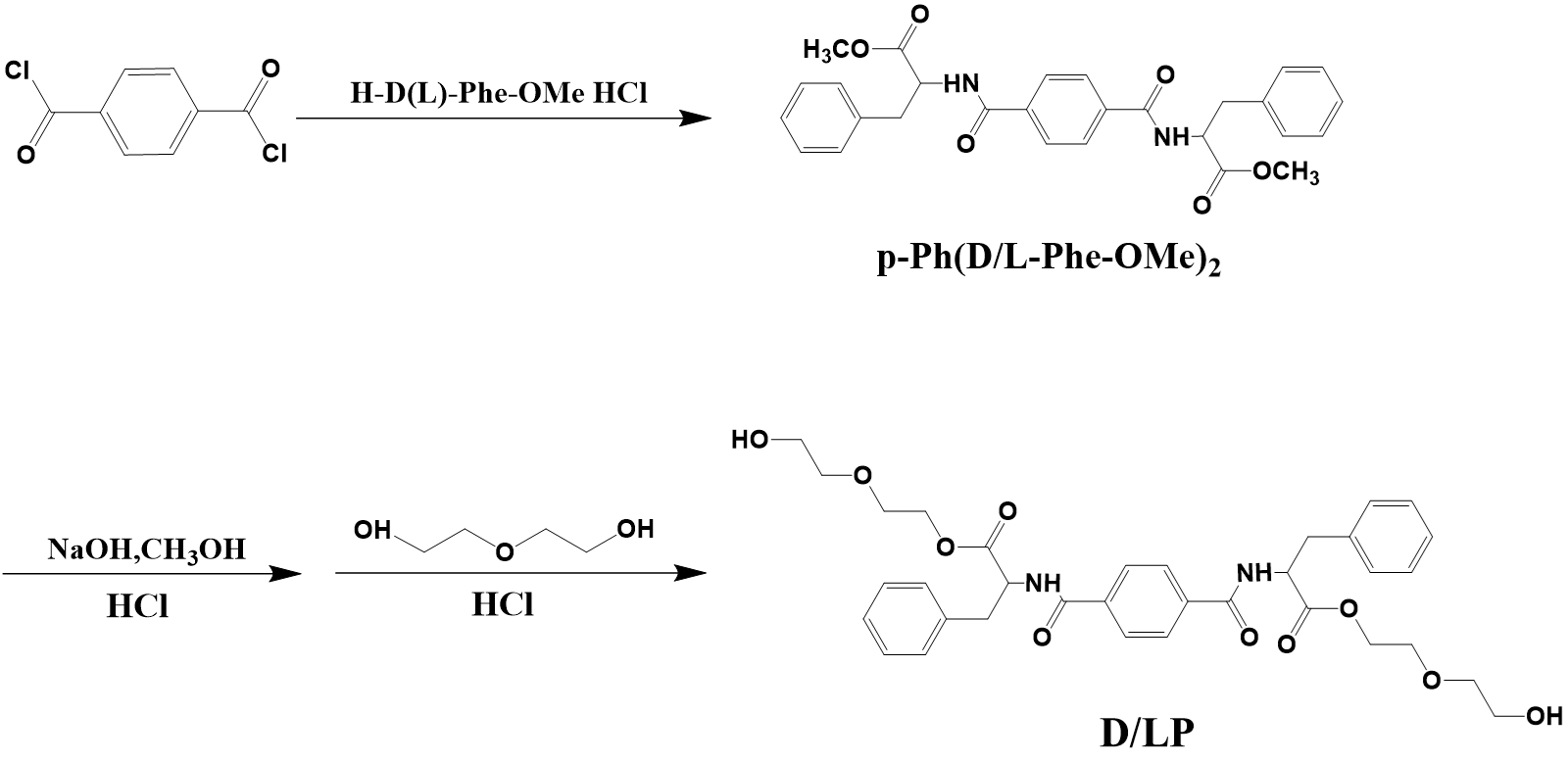
**

**Fig. S1** Synthesis procedures of L/DP. L/DP was synthesized by H-L-Phe-OMe and H-D-Phe-OMe, respectively


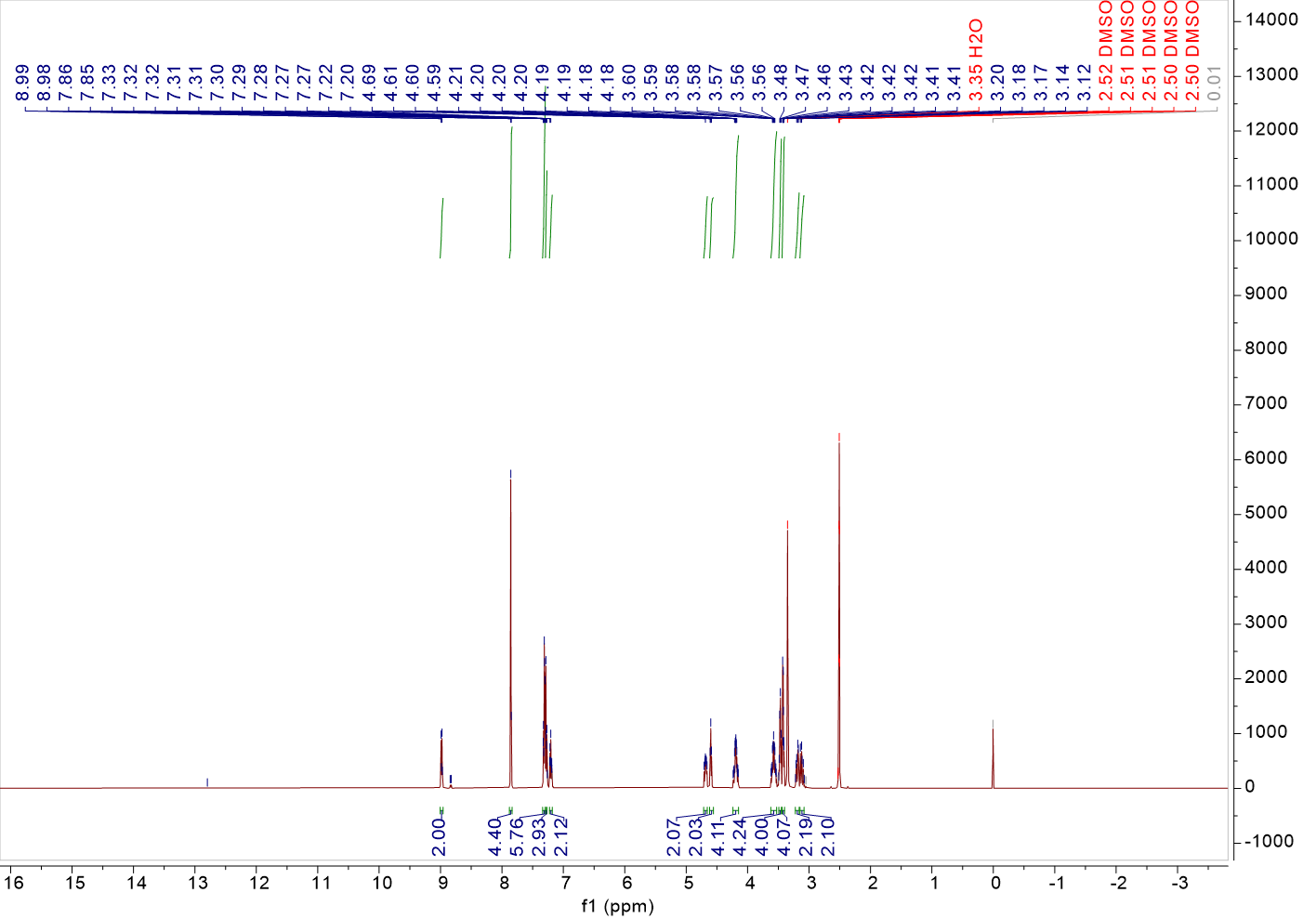


**Fig. S2** ^1^H-NMR spectrum of LP in DMSO-*d*6

^1^H NMR (500 MHz, DMSO-d6) δ 8.98 (d, J = 7.8 Hz, 2H), 7.85 (d, J = 3.4 Hz, 4H), 7.34 – 7.29 (m, 6H), 7.28 (d, J = 7.8 Hz, 3H), 7.23 – 7.18 (m, 2H), 4.68 (ddd, J = 10.0, 7.8, 5.4 Hz, 2H), 4.60 (t, J = 5.4 Hz, 2H), 4.19 (qdd, J = 12.0, 5.9, 3.7 Hz, 4H), 3.58 (tdd, J = 11.5, 8.7, 4.8 Hz, 4H), 3.47 (q, J = 5.2 Hz, 4H), 3.42 (dd, J = 5.5, 4.0 Hz, 4H), 3.19 (dd, J = 13.9, 5.3 Hz, 2H), 3.12 (dd, J = 13.8, 10.0 Hz, 2H).


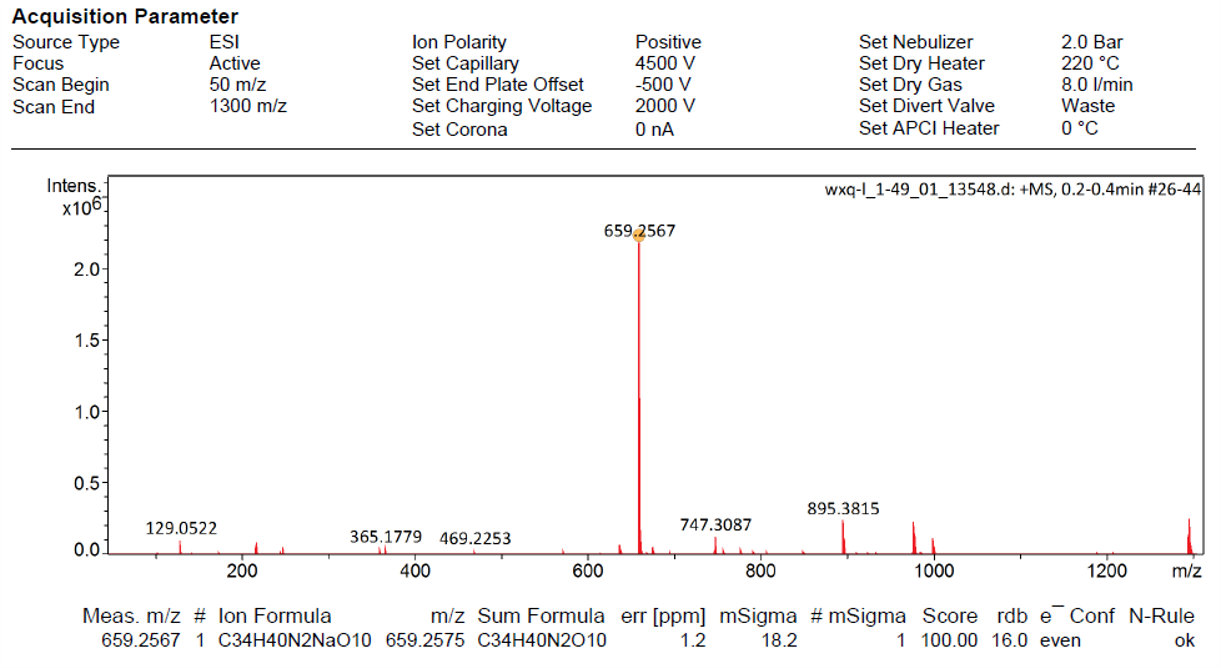


**Fig. S3** EI-HRMS spectrum of LP


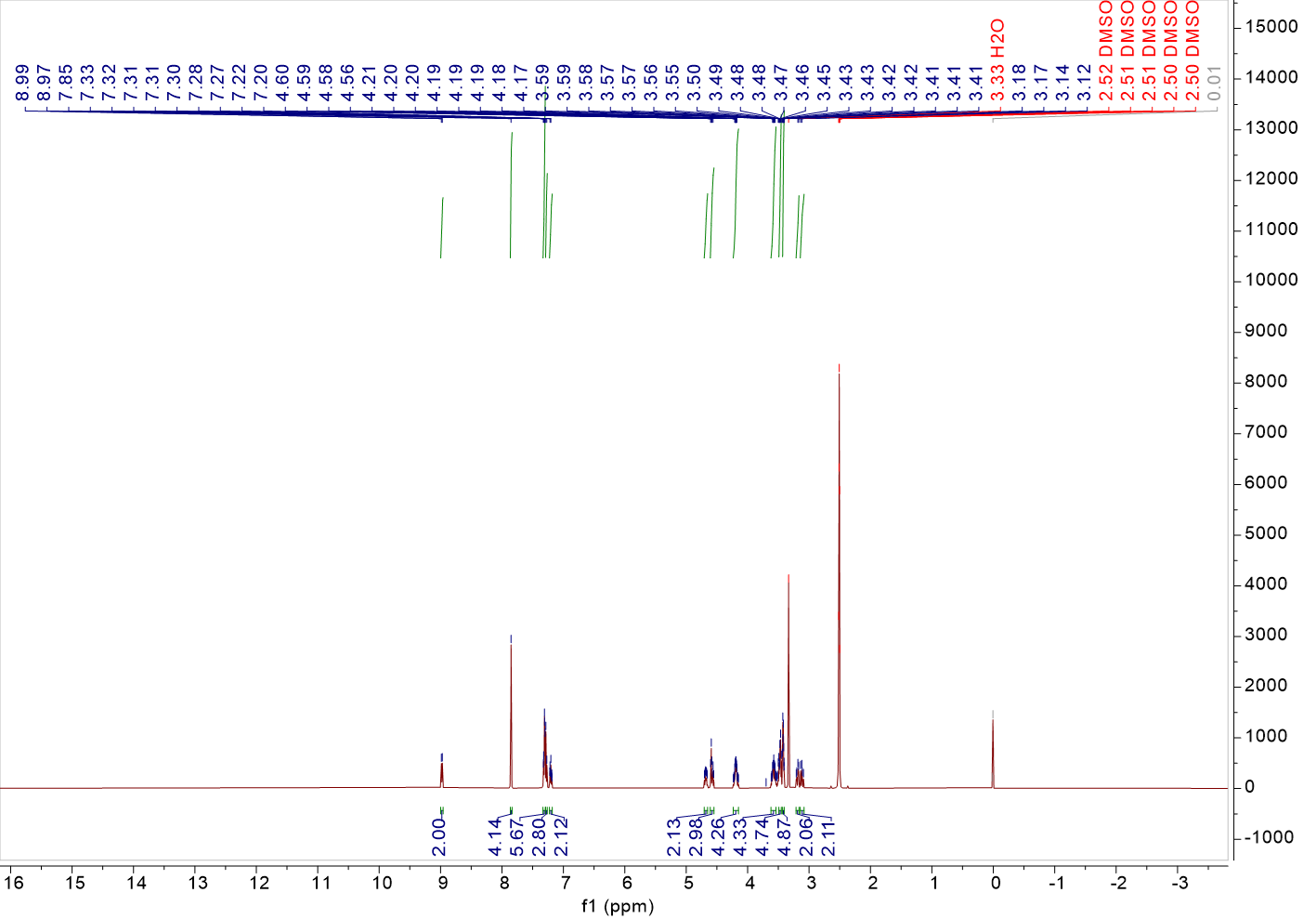


**Fig. S4** ^1^H-NMR spectrum of DP in DMSO-*d*6

^1^H NMR (500 MHz, DMSO-d6) δ 8.98 (d, J = 7.8 Hz, 2H), 7.85 (s, 4H), 7.33 – 7.29 (m, 6H), 7.28 (d, J = 7.5 Hz, 3H), 7.23 – 7.18 (m, 2H), 4.68 (ddd, J = 10.1, 7.8, 5.3 Hz, 2H), 4.58 (dt, J = 14.2, 5.5 Hz, 3H), 4.19 (qdd, J = 11.9, 6.0, 3.7 Hz, 4H), 3.58 (qdt, J = 7.7, 5.8, 2.9 Hz, 4H), 3.49 – 3.45 (m, 5H), 3.43 – 3.40 (m, 5H), 3.19 (dd, J = 13.8, 5.3 Hz, 2H), 3.11 (dd, J = 13.8, 10.0 Hz, 2H).


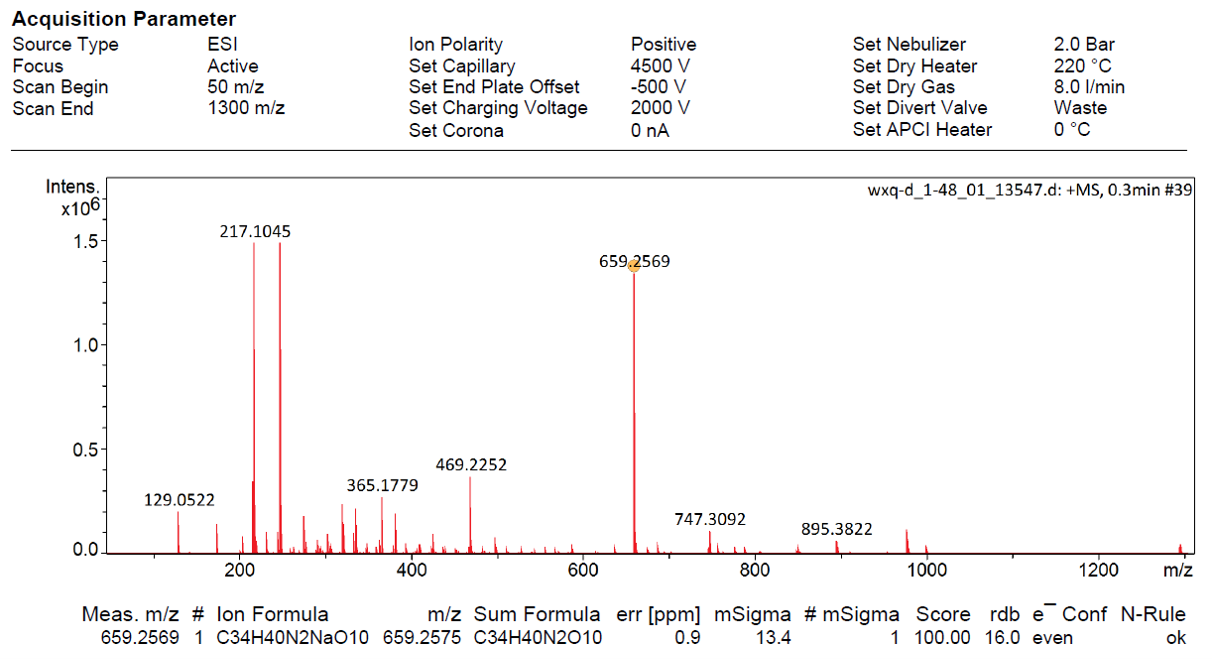


**Fig. S5** EI-HRMS spectrum of DP

**
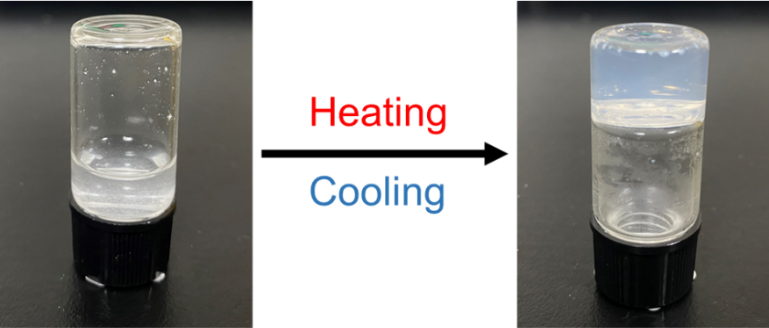
**

**Fig. S6** Photographs of LP before and after a heating and cooling process

**
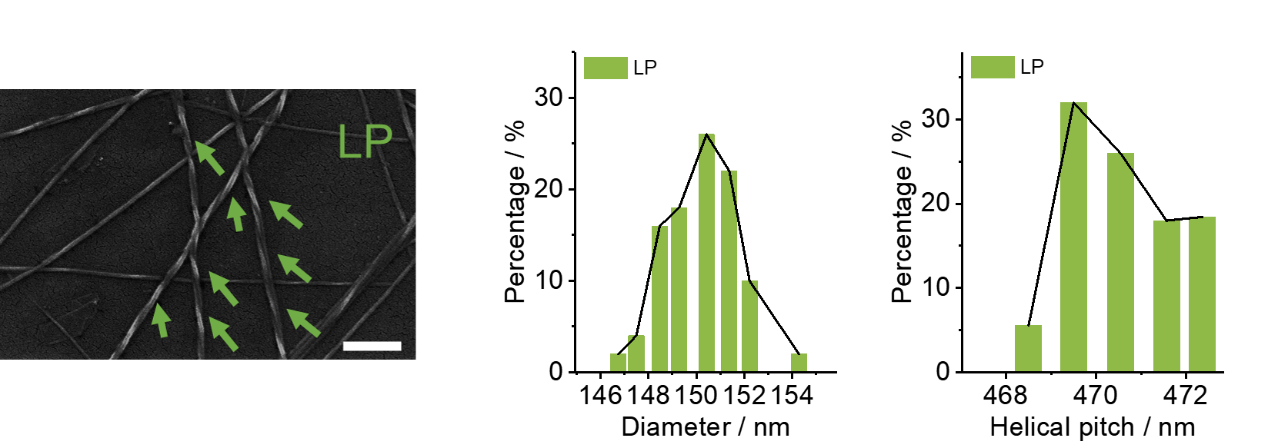
**

**Fig. S7** SEM image of LP and the statistical data of diameter and helical pitch (n = 50). Scale bar: 500 nm


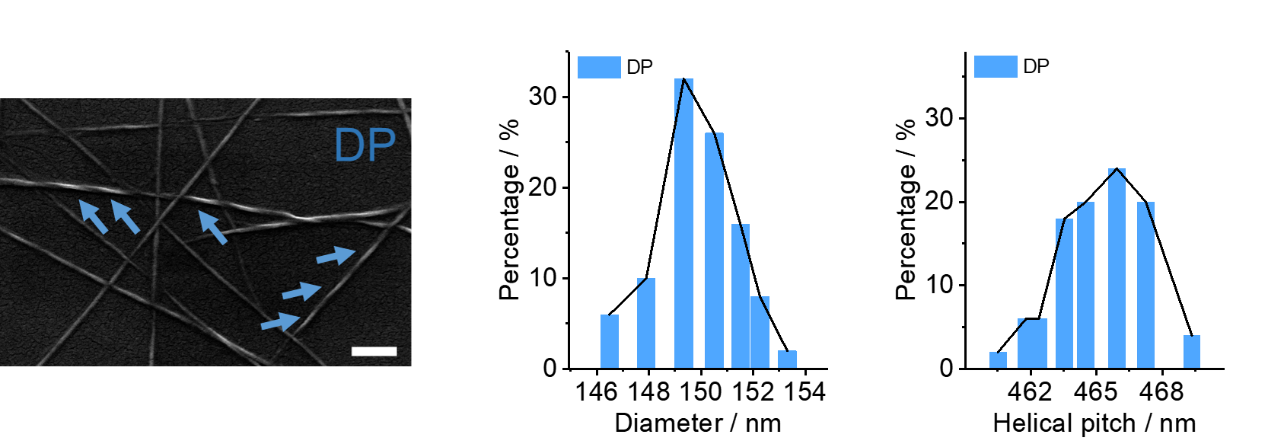


**Fig. S8** SEM image of DP and the statistical data of diameter and helical pitch (n = 50). Scale bar: 500 nm


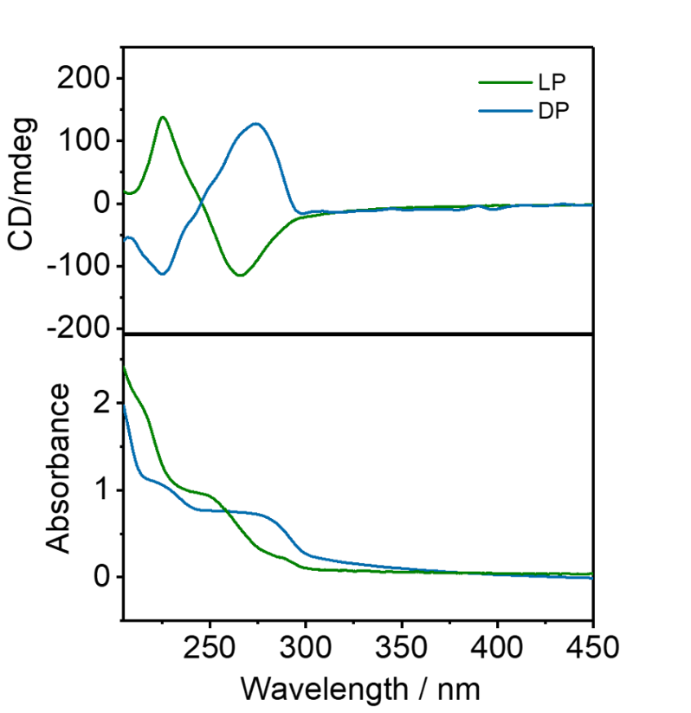


**Fig. S9** CD and corresponding UV-vis spectra of LP and DP


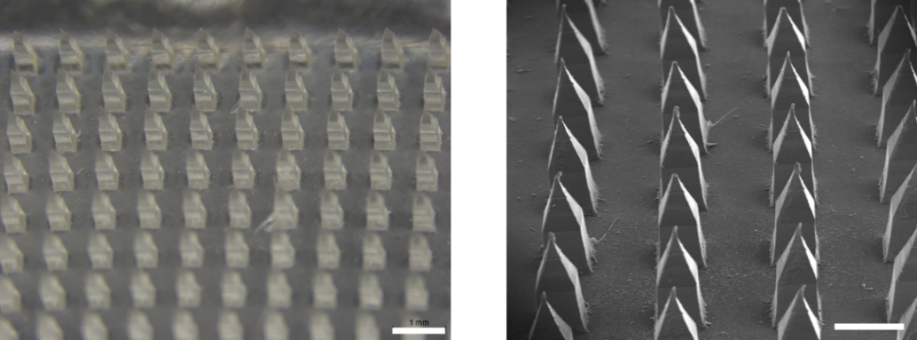


**Fig. S10** Stereoscopic and SEM image of D-HA MNs. Scale bar (left): 1 mm and scale bar (right): 500 μm


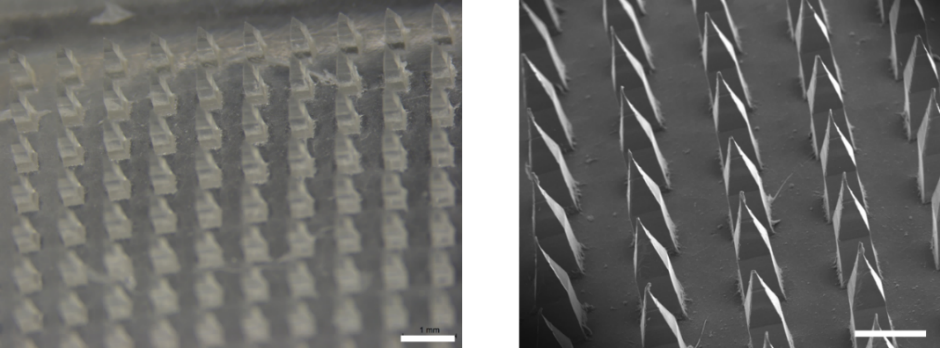


**Fig. S11** Stereoscopic and SEM image of HA MNs. Scale bar (left): 1 mm and scale bar (right): 500 μm

**
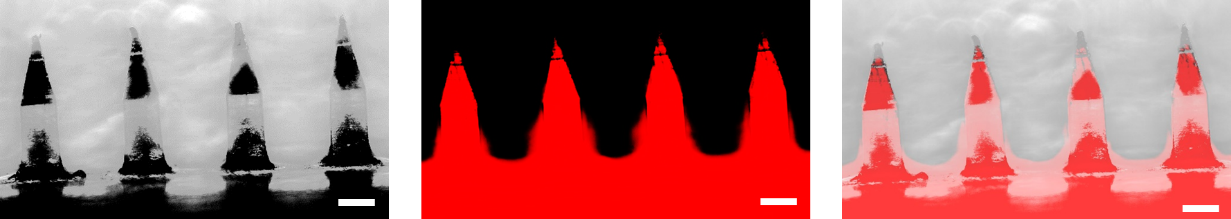
**

**Fig. S12** Representative fluorescence images of the Rhodamine B labeled L-HA MNs. Scale bar, 200 μm


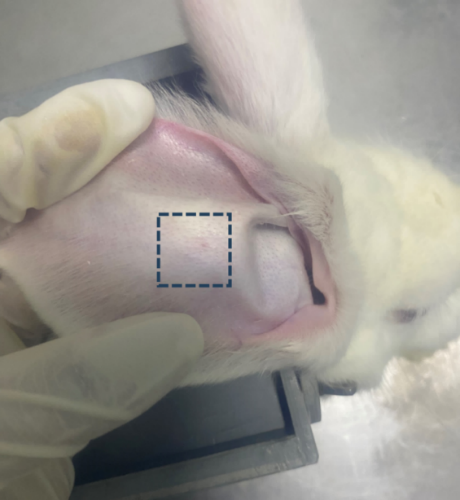


**Fig. S13** Image of rabbit ear after microneedle treatment

**Fig. S14** Cell viability of NSFs with different concentrations of L/DP


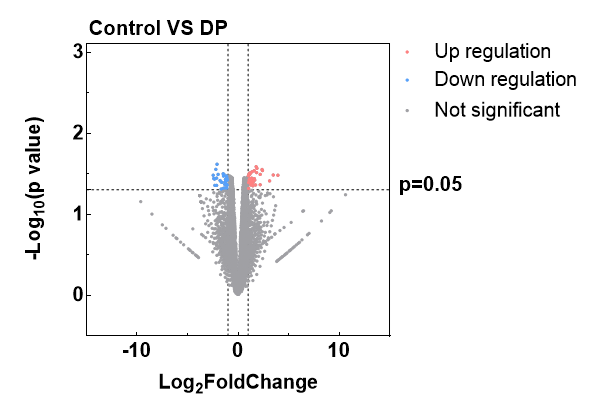


**Fig. S15** Volcano plot of differentially expressed genes in control vs 3% DP


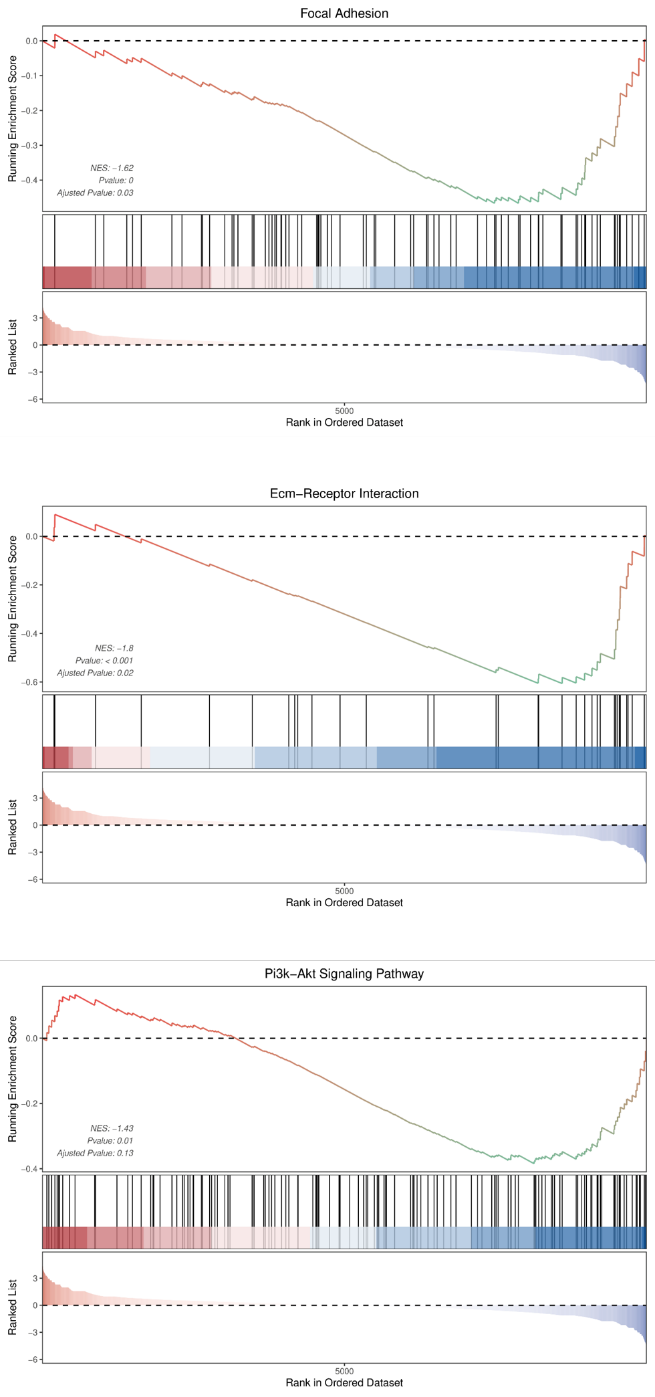


**Fig. S16** Gene set enrichment analysis (GSEA) of focal adhesion and ECM-receptor interaction in control vs. LP

**
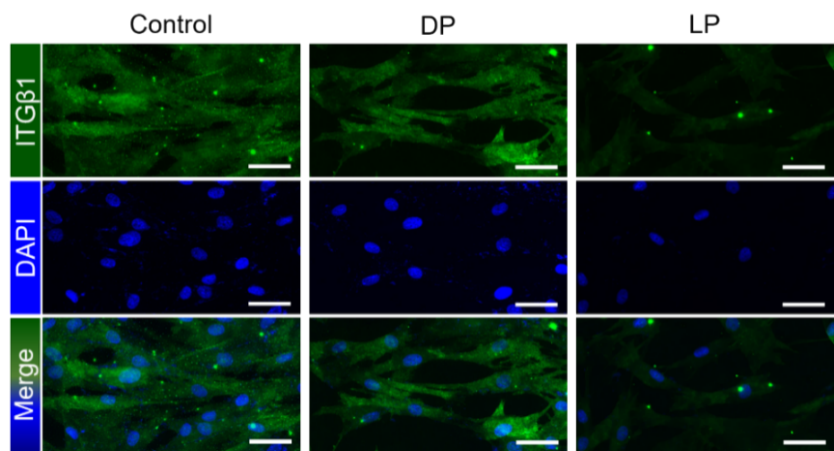
**

**Fig. S17** Immunofluorescent staining images of ITGβ1 after DP and LP treatments. Green (ITGβ1) and blue (DAPI). Scale bar: 40 μm


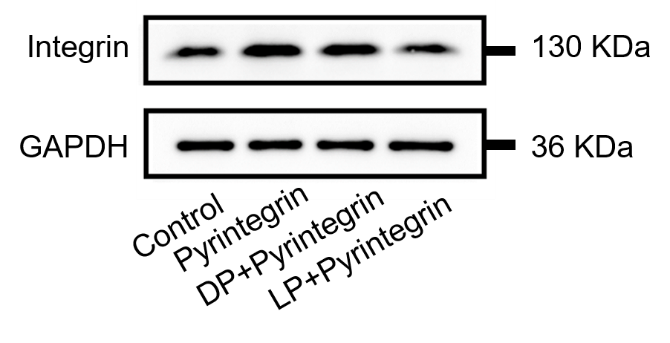


**Fig. S18** The WB images of control, pyrintegrin, DP+pyrintegrin, and LP+pyrintegrin


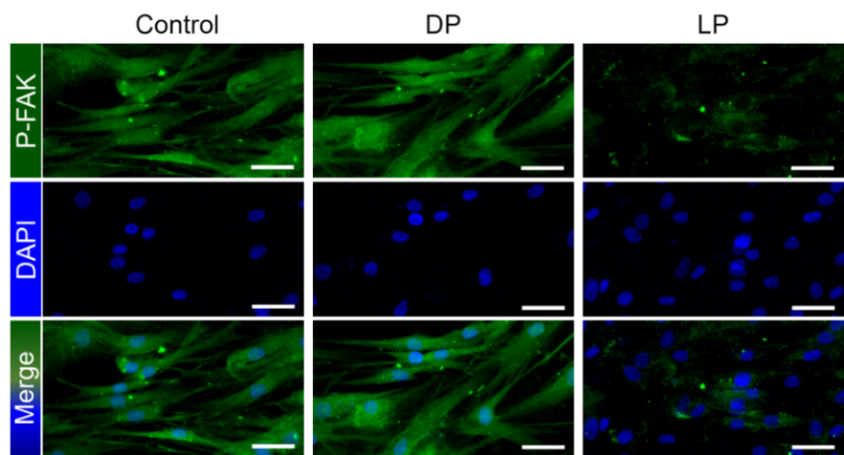


**Fig. S19** Immunofluorescent staining images of p-FAK after DP and LP treatments. Green (p-FAK) and blue (DAPI). Scale bar: 40 μm


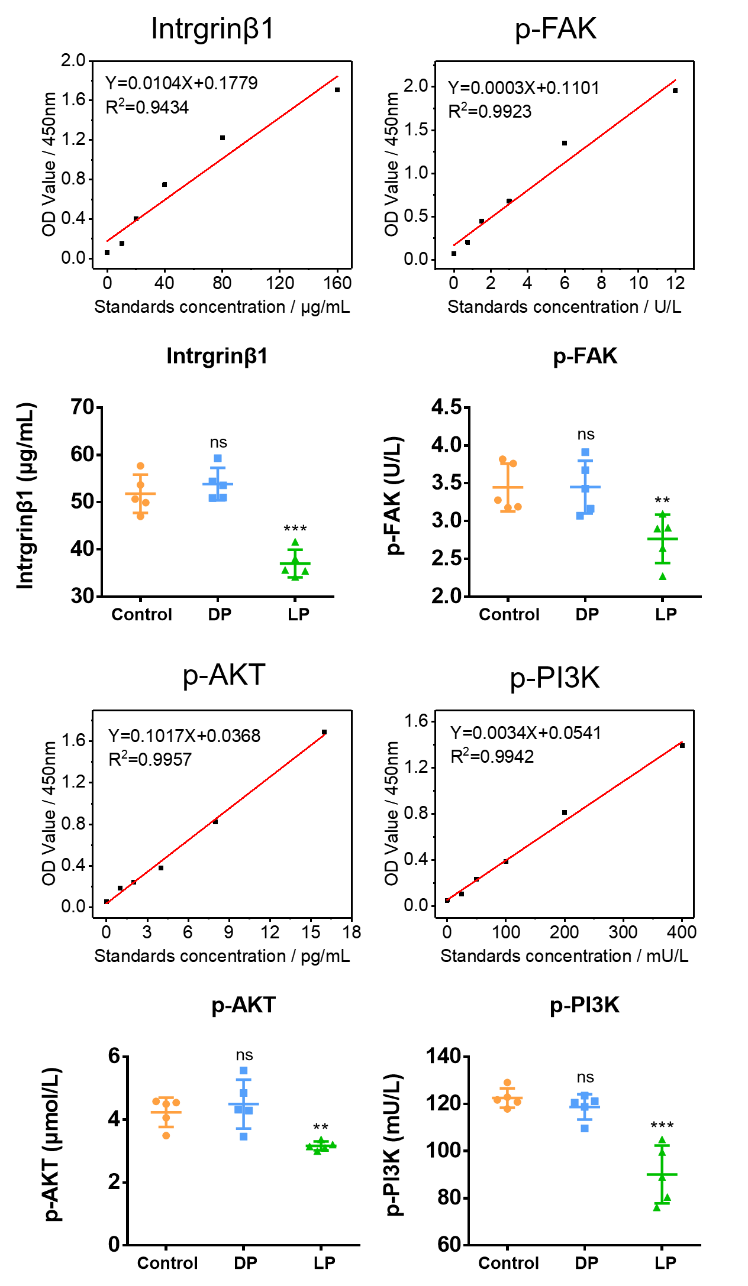


**Fig. S20** The Elisa experiment results of ITGβ1 and p-FAK in control, 3% DP, and 3% LP (n=5 independent samples)


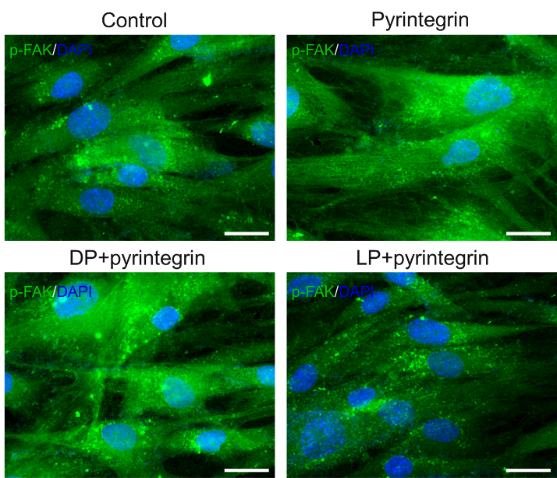


**Fig. S21** Immunofluorescent staining images of p-FAK after various treatments. Green (p-FAK) and blue (DAPI). Scale bar: 40 μm


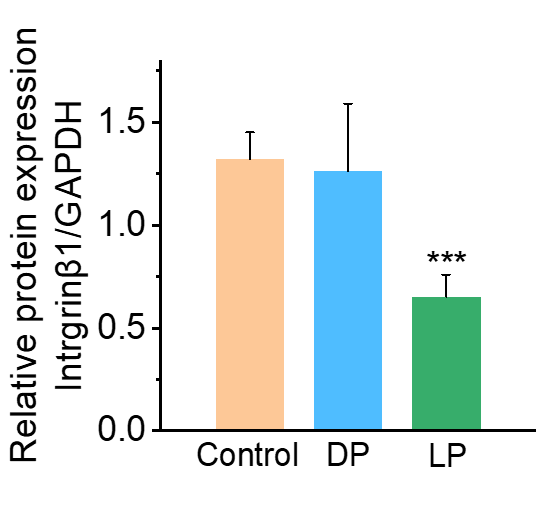


**Fig. S22** Quantitative analyses of the protein expression levels of ITGβ1 in different groups (n=3 independent samples)


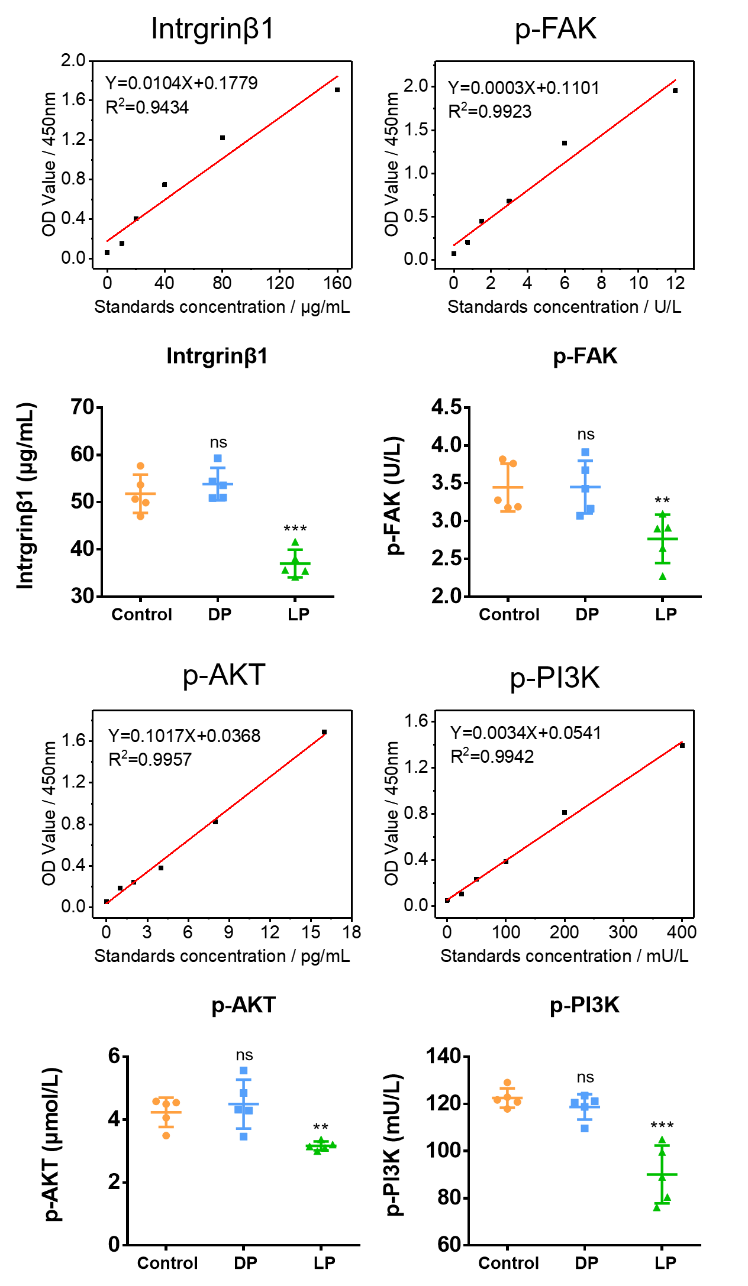


**Fig. S23** The Elisa experiment results of p-AKT and p-PI3K in control, 3% DP, and 3% LP


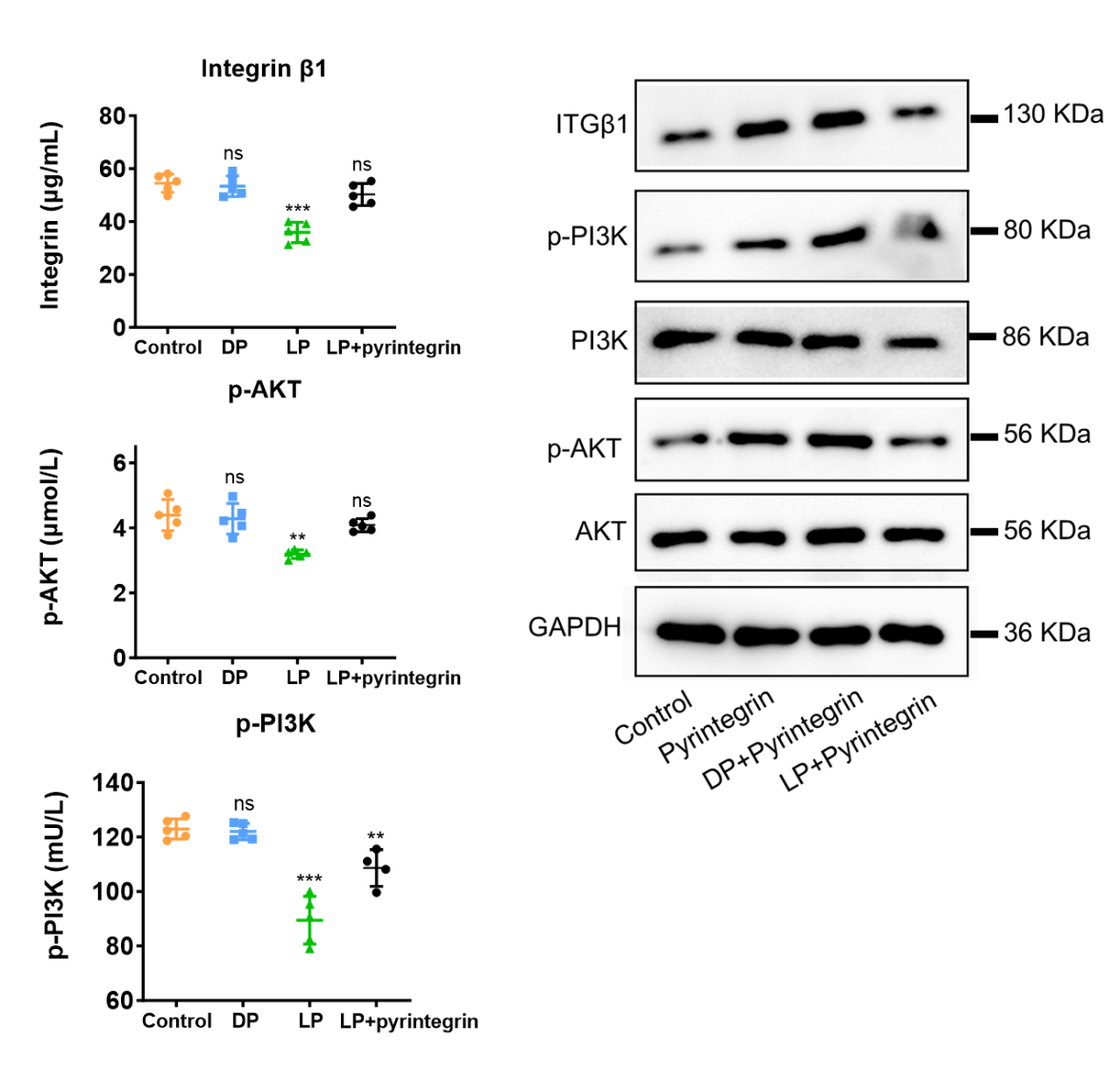


**Fig. S24** The Elisa experiment and WB results of ITGβ1, p-AKT, and p-PI3K after various treatments (n=5 independent samples)


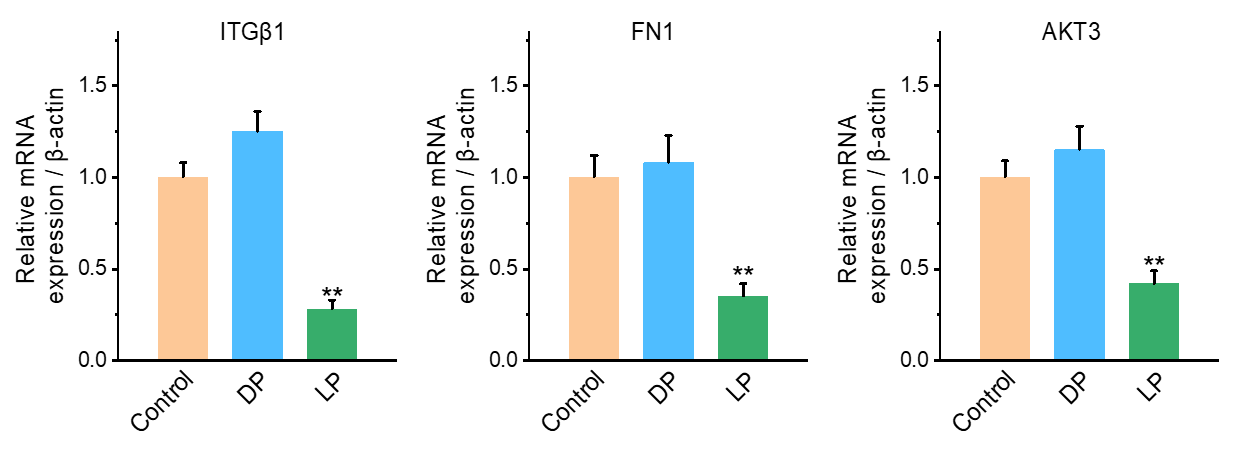


**Fig. S25** Quantitative analyses of the relative mRNA expression levels of ITGβ1, FN1, and AKT3


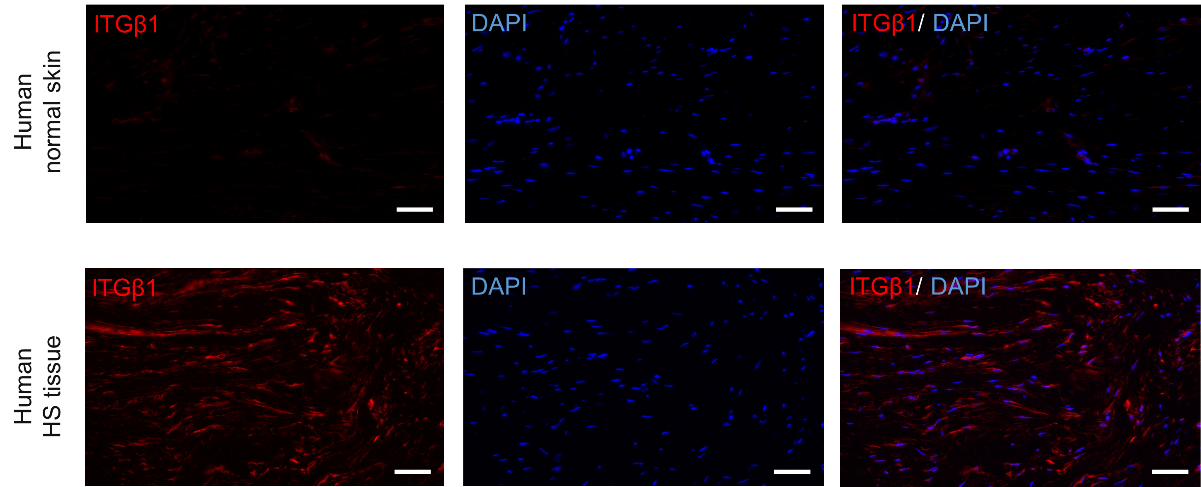


**Fig. S26** Representative immunofluorescence staining of ITGβ1 in human normal skin and human HS tissue. Scale bar, 20 μm


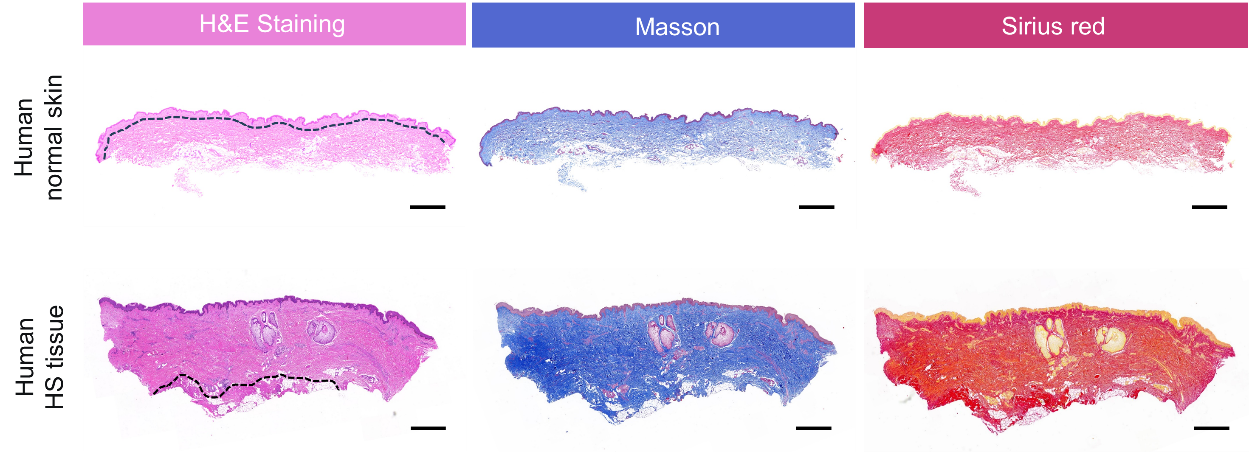


**Fig. S27** Representative H&E staining, Masson staining, and Sirius red staining images of human normal skin and human HS tissue. Scale bar, 1000 μm


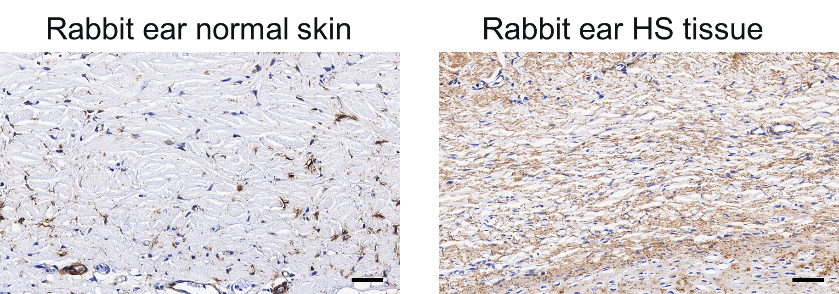


**Fig. S28** Representative immunohistochemistry staining of ITGβ1 in rabbit ear normal skin and rabbit ear HS tissue. Scale bar, 1000 μm


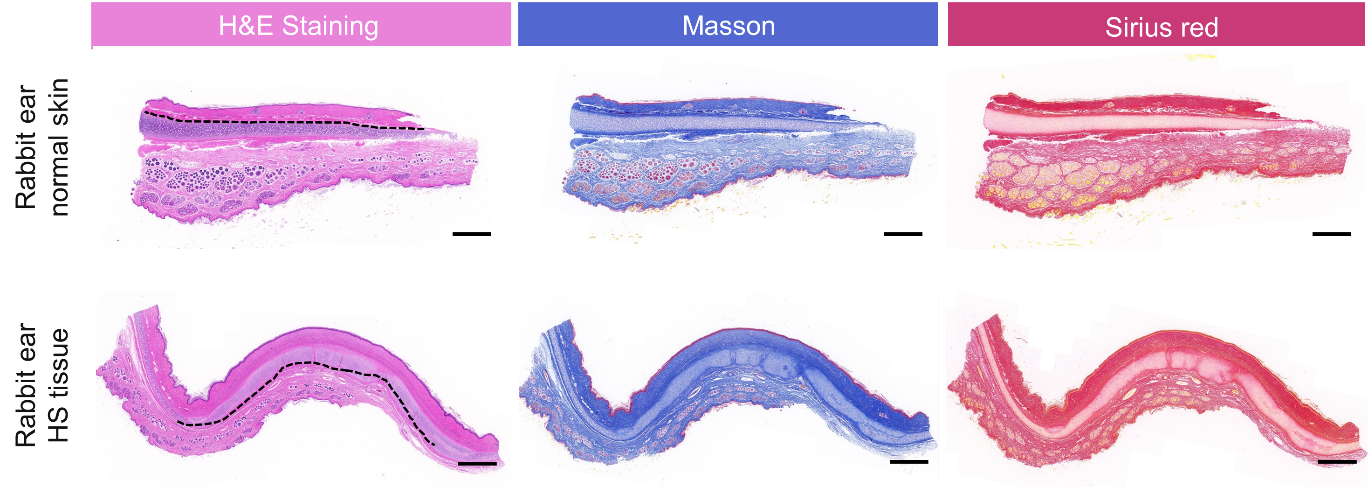


**Fig. S29** Representative H&E staining, Masson staining, and Sirius red staining images of rabbit ear normal skin and rabbit ear HS tissue. Scale bar, 1000 μm


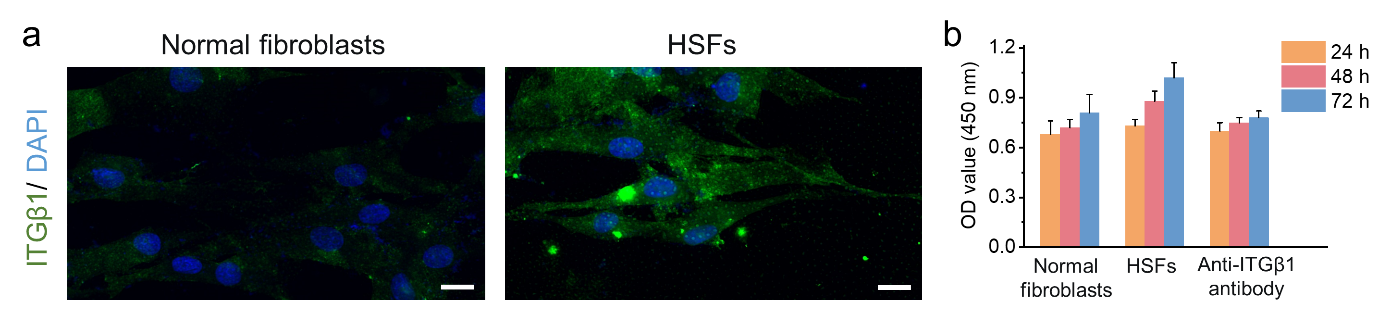


**Fig. S30** **a** Representative immunofluorescence images of ITGβ1 (green) and DAPI (blue) in normal fibroblasts and HSFs. Scale bar, 50 μm. **b** The absorbance value at 450 nm in various groups for 24, 48, and 72 hours (n=3 independent samples)


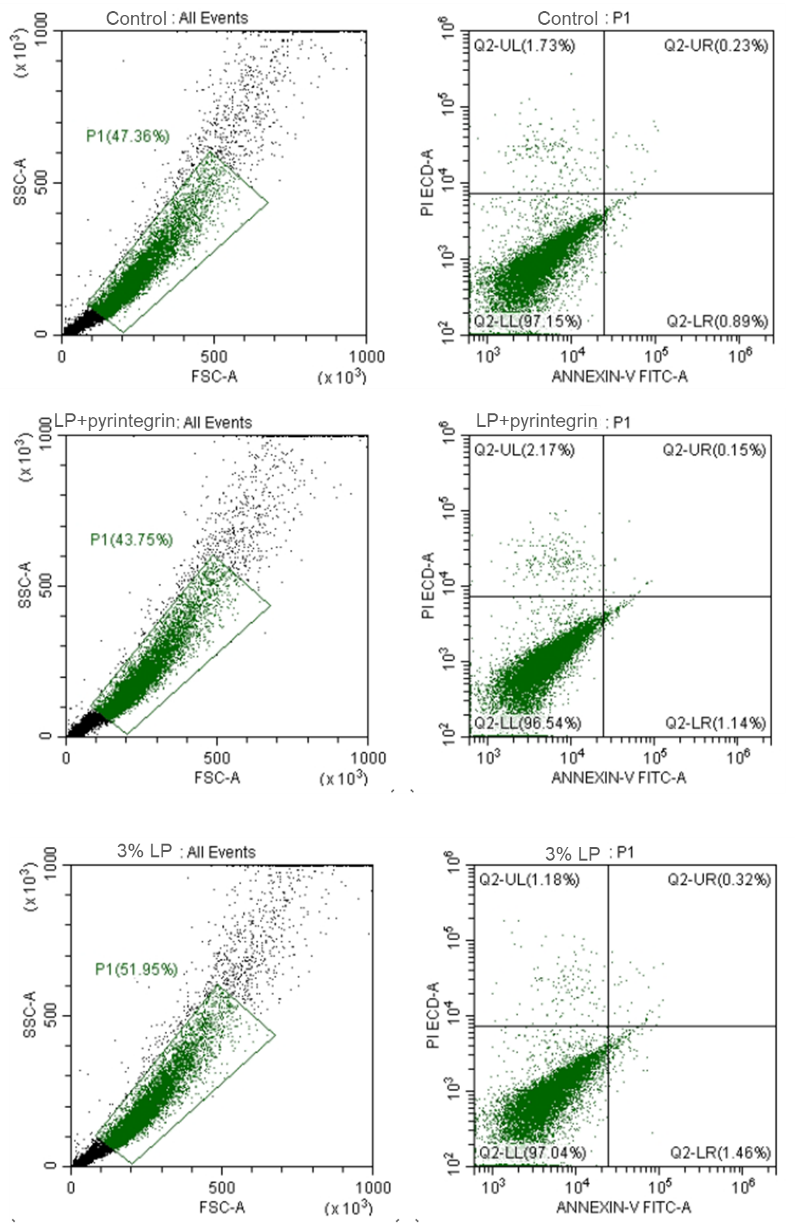


**Fig. S31** The flow cytometry analysis after various treatments


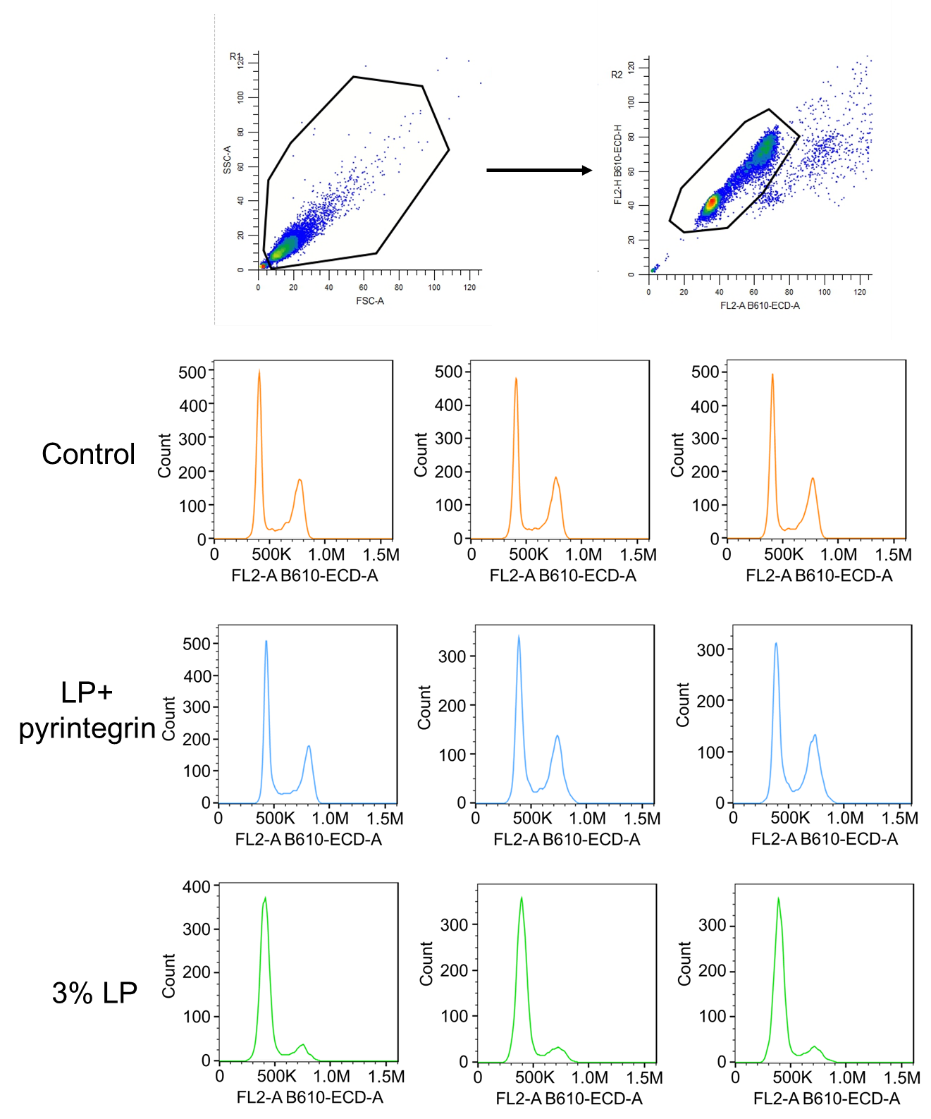


**Fig. S32** The cell cycle analysis of different treatments


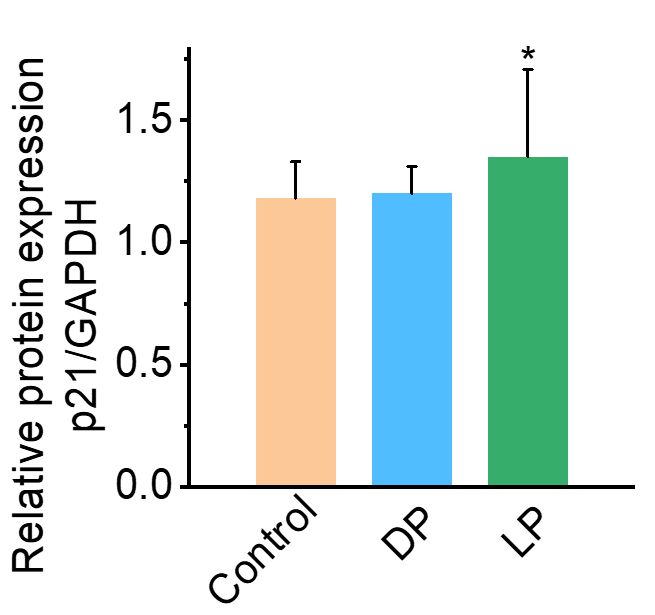


**Fig. S33** Relative protein expression of p21 after various treatments (n=3 independent samples)


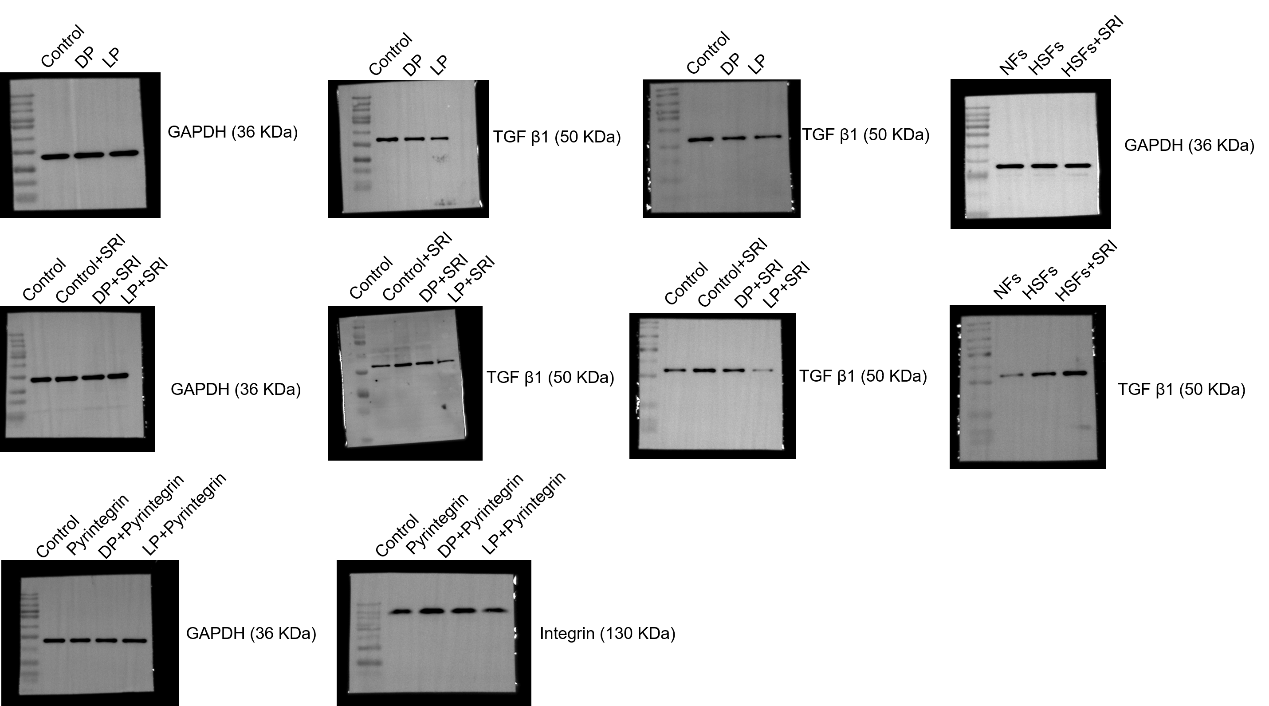


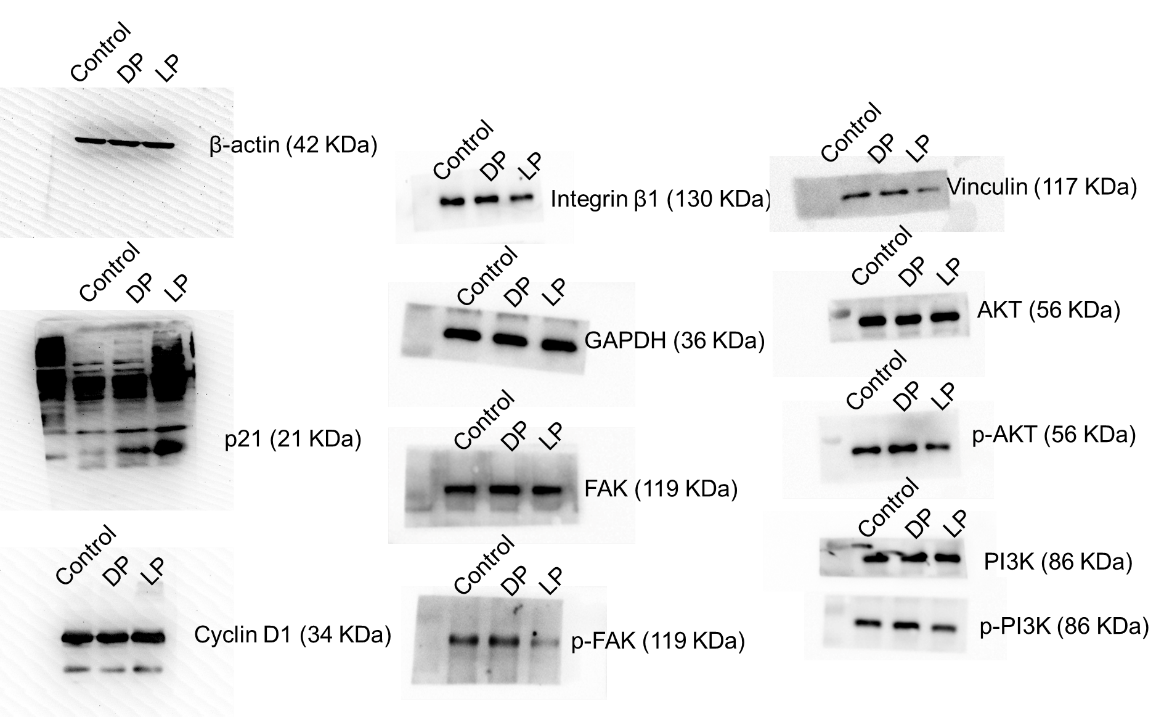


**Fig. S34** The WB images of different treatments


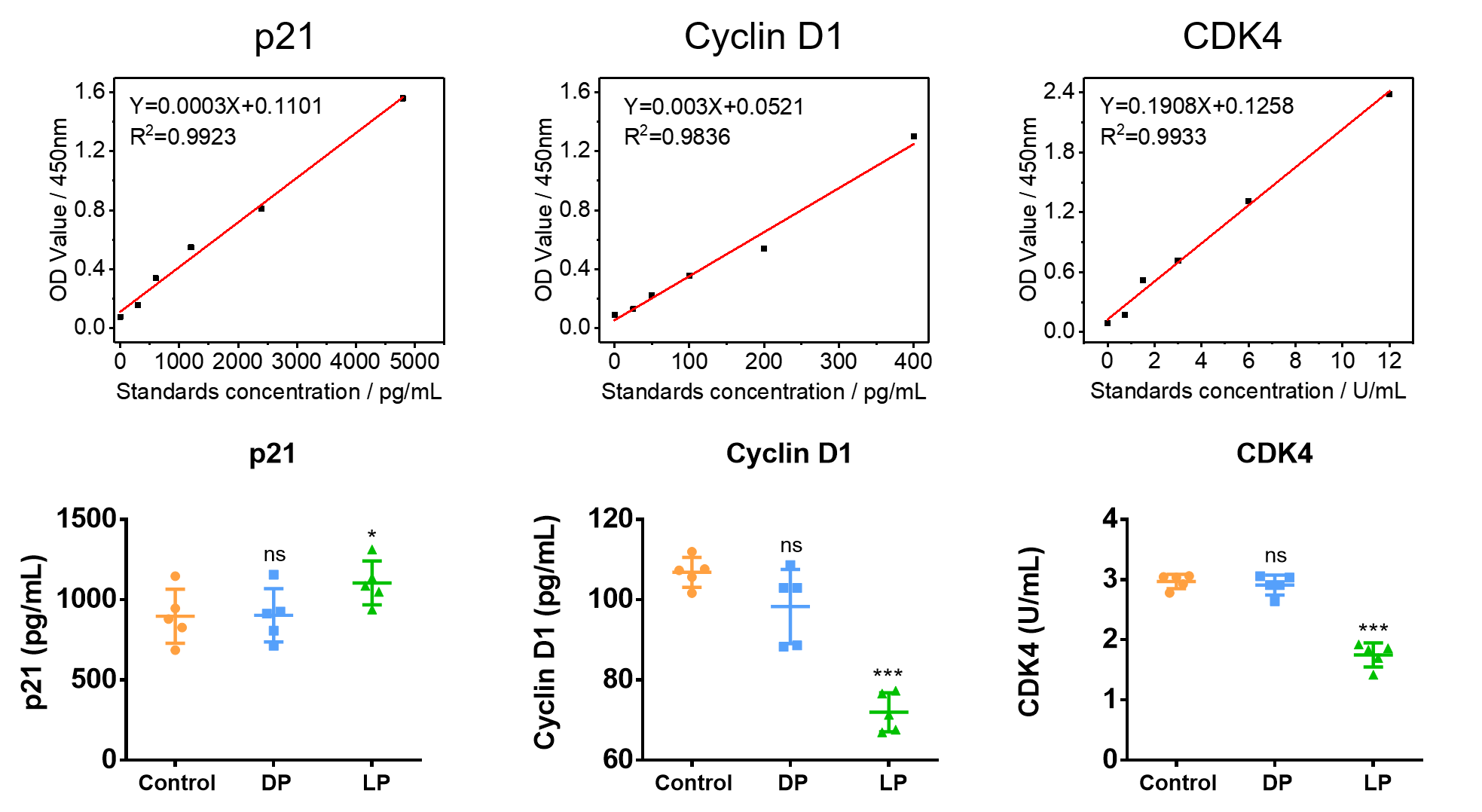


**Fig. S35** The Elisa experiment results of p21 and cyclin D1 in control, DP, and LP (n=5 independent samples)


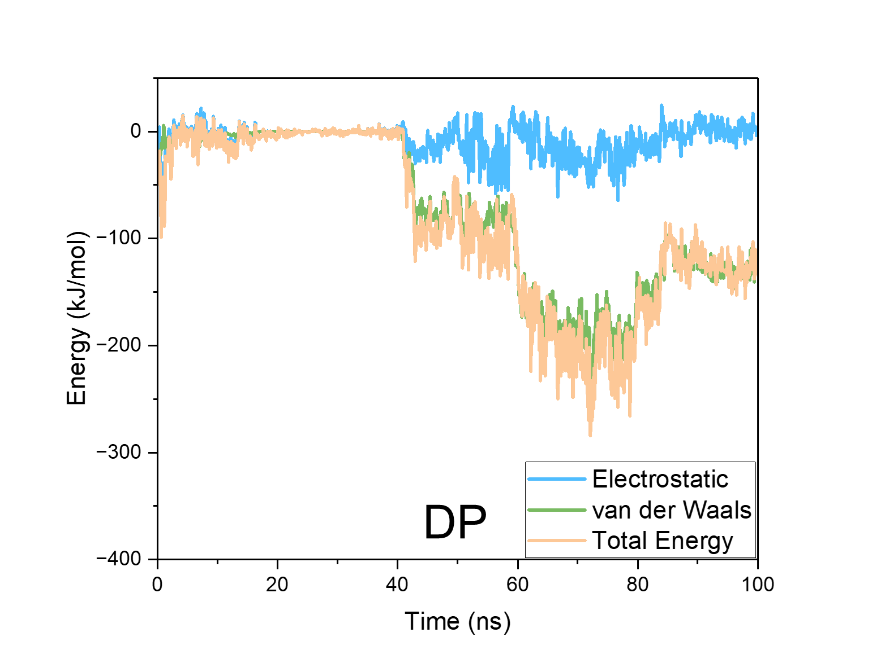


**Fig. S36** The interaction energy between the protein and the substrate changes over the simulation time period


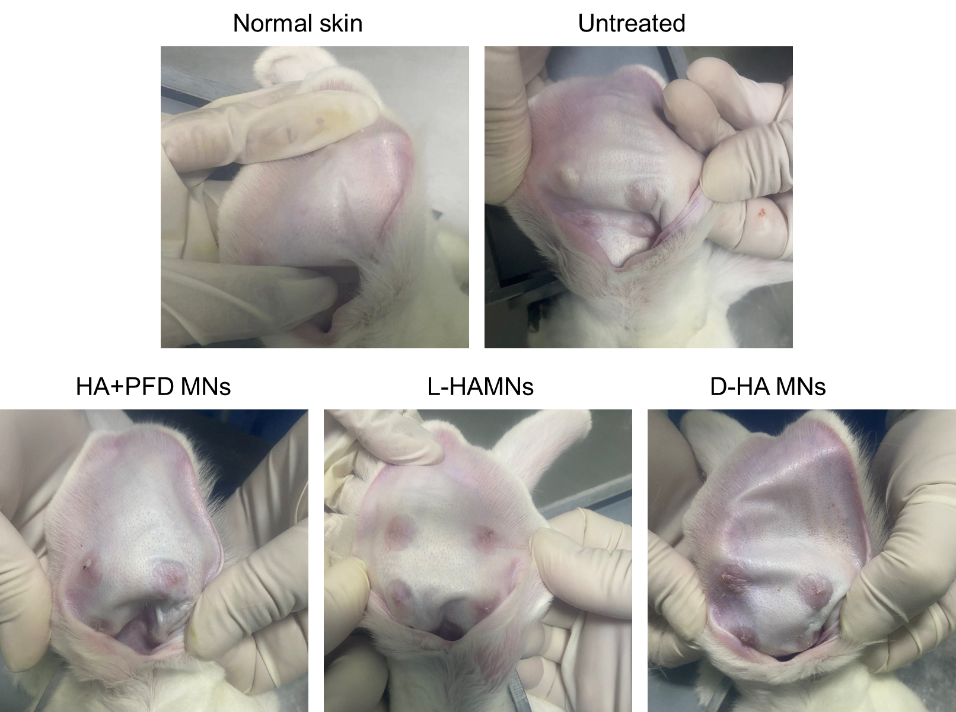


**Fig. S37** Image of rabbit ear scar tissue with different treatments after 30 days


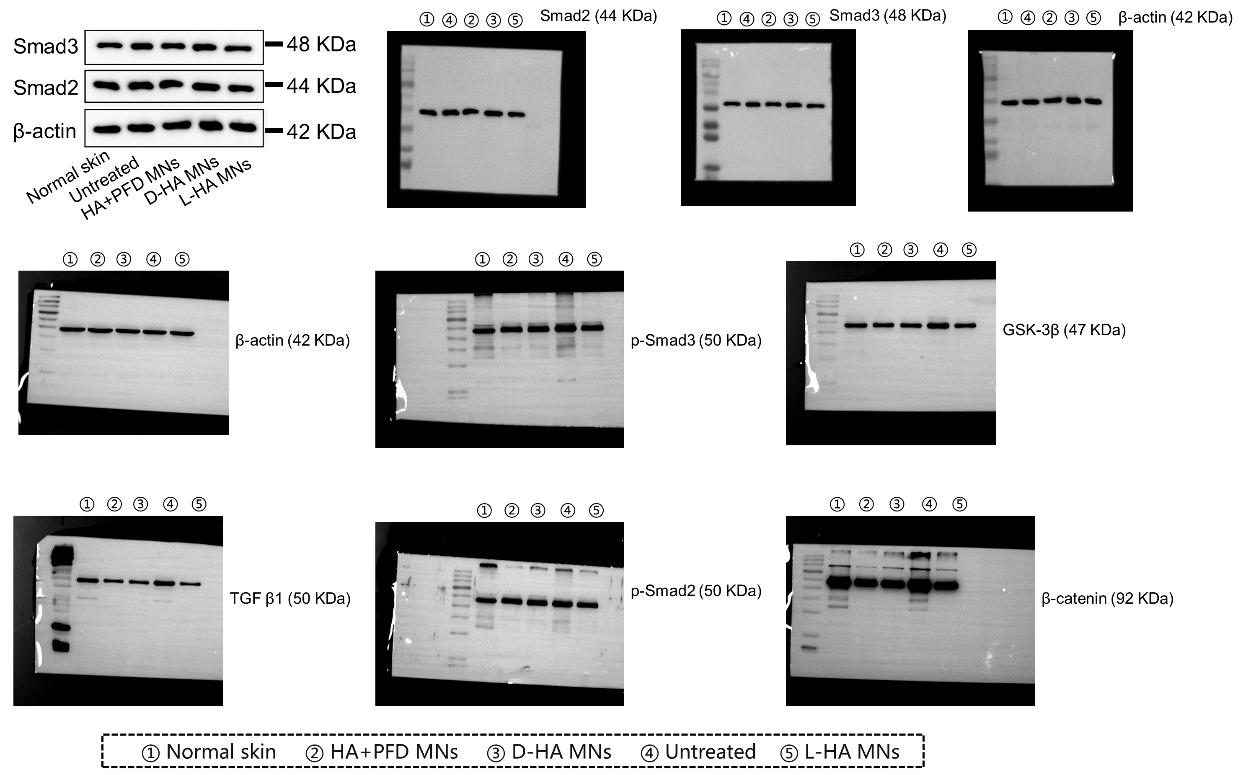


**Fig. S38** Western blot analysis results of β-catenin, p-Smad2, p-Smad3, TGF β1, and GSK-3β with various treatments. β-actin was used as the protein loading control

**Fig. S39** Relative protein expression of p-Smad2 after various treatments (n=3 independent samples)


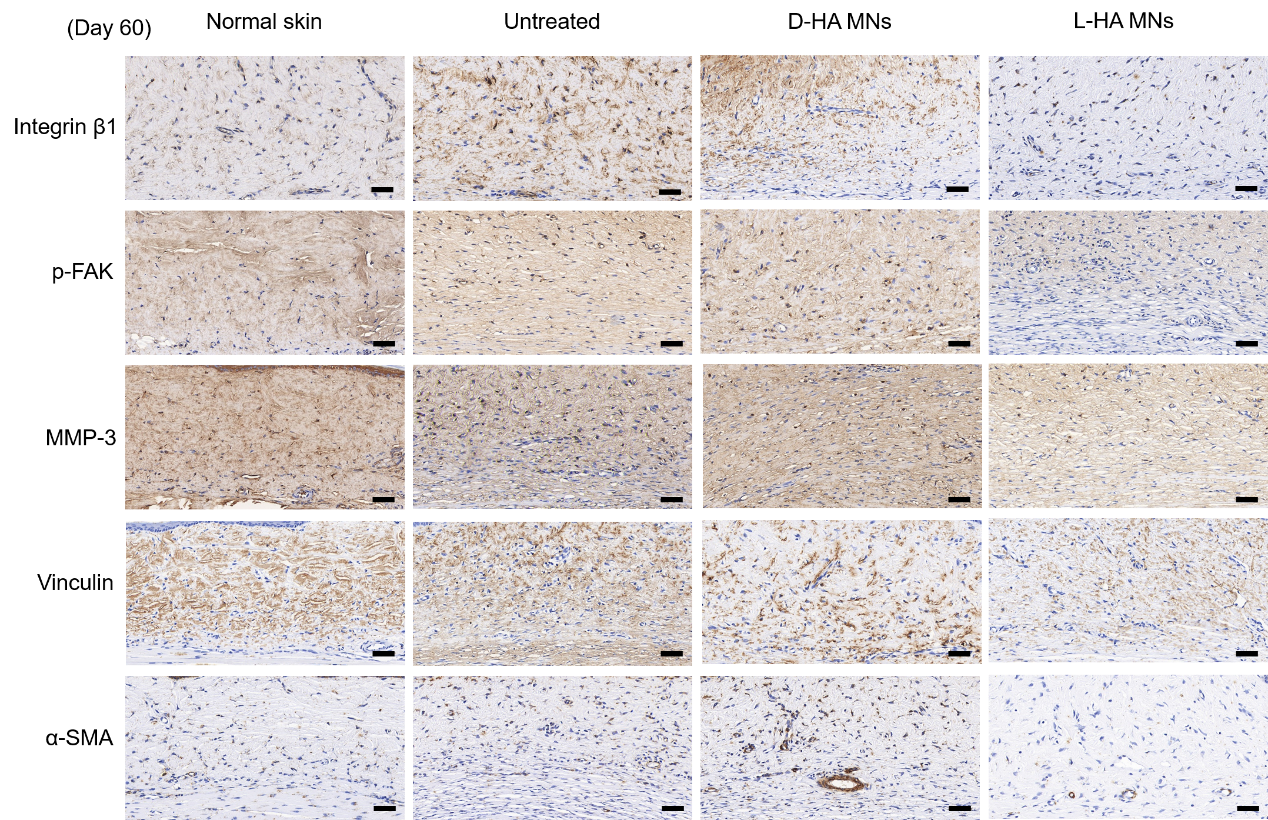


**Fig. S40** Immunohistochemical images of scar tissues stained with p-FAK, MMP-3, Vinculin, and α-SMA in various groups. Scale bar, 100 μm


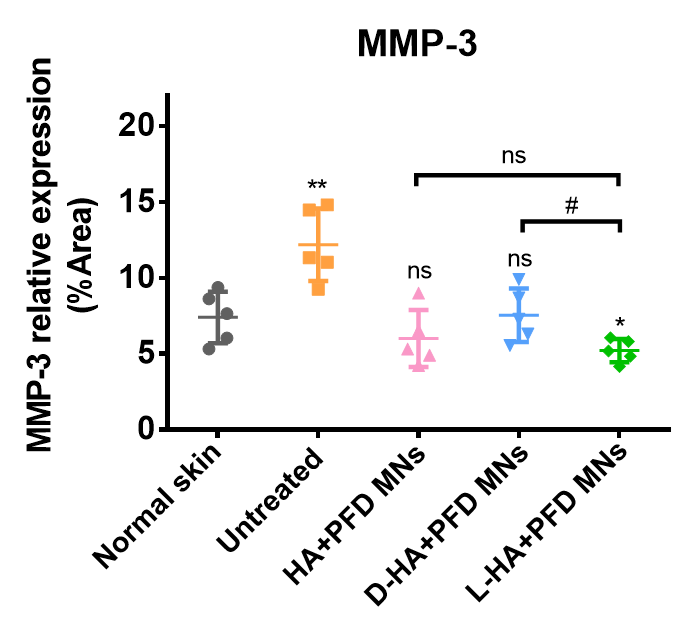


**Fig. S41** Relative expression of MMP-3 after various treatments (n=3 independent samples)
